# A structural equation modeling approach for the association of a healthy eating index with metabolic syndrome and cardio-metabolic risk factors among obese individuals

**DOI:** 10.1371/journal.pone.0219193

**Published:** 2019-07-01

**Authors:** Mahdieh Khodarahmi, Mohammad Asghari-Jafarabadi, Mahdieh Abbasalizad Farhangi

**Affiliations:** 1 Nutrition Research Center, Department of Community Nutrition, Faculty of Nutrition and Food Science, Tabriz University of Medical Sciences, Tabriz, Iran; 2 Student Research Committee, Department of Nutrition, Faculty of Nutrition and Food Science, Tabriz University of Medical Sciences, Tabriz, Iran; 3 Road Traffic Injury Research Center, Tabriz University of Medical Sciences, Tabriz, Iran; 4 Department of Statistics and Epidemiology, Faculty of Health, Tabriz University of Medical Sciences, Tabriz, Iran; 5 Drug Applied Research Center, Tabriz University of Medical Sciences, Tabriz, Iran; Monash University, AUSTRALIA

## Abstract

**Background:**

Numerous studies have evaluated the association between dietary factors and cardiovascular risk among patients with chronic disease. It is worthwhile to assess these associations in a combination model rather than in an isolated form. In the current study, we aimed to use structural equation modeling (SEM) to assess the association of adherence to a healthy eating index (HEI)-2015 with socio-demographic factors, psychological characteristics, metabolic syndrome (MetS) and other cardio-metabolic risk factors among obese individuals.

**Methods:**

This cross-sectional study was conducted among 188 healthy obese adults (96 males and 92 females) aged 20–50 years in Tabriz. A validated semi-quantitative food frequency questionnaire (FFQ) was used to record dietary intake and to estimate HEI-2015. Anthropometric parameters, blood pressure and biochemical measurements were evaluated according to standard protocols. Interrelationships among socio-demographic parameters and HEI with cardio-metabolic risk factors were analyzed using SEM.

**Results:**

The results of SEM analysis revealed that HEI mediated the association between age and several cardio-metabolic risk factors including fat mass (FM), fat free mass (FFM), systolic blood pressure (SBP) and high-density lipoprotein (HDL) (p < 0.05). Moreover, adherence to Dietary Guidelines for Americans (DGA) appears to mediate association between gender and waist circumference (B = -9.78), SBP (B = -4.83), triglyceride (B = -13.01) and HDL (B = 4.31). HEI also mediated indirect negative effects of socioeconomic status on FM (B = -0.56), FFM (B = -0.25), SBP (B = -0.55) and diastolic blood pressure (DBP) (B = -0.3). Additionally, depression and age had indirect unfavorable effects on some insulin resistance indices such as homeostasis model assessment of insulin resistance (B = 0.07; p<0.05, for age) and quantitative insulin sensitivity check index (p<0.05, for age and depression) via HEI. High adherence to HEI was found to be inversely associated with MetS risk (p<0.05).

**Conclusion:**

Adherence to HEI-2015 seems to mediate the effect of socio-demographic parameters and mental health on cardio-metabolic risk factors as well as MetS risk. Further studies are needed to confirm these findings.

## Introduction

Obesity is a major public health problem worldwide and its prevalence has increased dramatically in both developed and developing countries during recent years [1, 2]. According to a World Health Organization (WHO) report, in 2016 over 650 million adults were obese throughout the world. Obesity is also an important health problem in Iran, with a prevalence of about 22%, or almost 1 in 4, among the adult population [3]. The evidence shows that obesity increases the chance of many chronic diseases, including cardiovascular disorders, Type 2 Diabetes, respiratory disorders, several musculoskeletal problems, and certain types of cancers [4]. Furthermore, obese individuals are at increased risk of developing dyslipidemia, hypertension, insulin resistance, and metabolic syndrome (MetS) [5]. The etiology of obesity is multifactorial: numerous factors, including genetic and life-style related parameters such as nutrition and socio-economic status (SES), play a crucial role in its development [6, 7]. Recently it has been suggested that studying single nutrients or food items and their contribution may not give a good representative image of diet-disease associations, while it may be better understood in a combined analysis of food and nutrients [8]. Dietary quality indices are useful tools for evaluating overall dietary patterns and studying diet-disease associations. The Healthy Eating Index (HEI), first developed by the U.S. Department of Agriculture (USDA), evaluates adherence to Dietary Guidelines for Americans (DGA) [9]. These guidelines, which provide evidence-based recommendations for major chronic diseases, are updated every five years [10]. HEI-2015 is the latest version, reflecting dietary guidelines for 2015 through 2020 [10]. It has been reported that prior versions, including HEI–2010 and HEI-2005, are related to reduced risks of all-cause mortality, cardiovascular disease, cancer, and Type 2 diabetes [11]. However, there are some inconsistencies regarding the association between HEI and obesity [12] and the results to date are ambiguous [13]. Additionally, the efficacy of the latest version of HEI (HEI-2015) in obesity has not yet been evaluated. According to the results of prior studies, MetS prevalence is increasing concurrent with the growing prevalence of obesity among Iranian adults [14]. On the other hand, it has been shown that the quality of the Iranian diet needs to improve [15]. Moreover, investigating the association between diet quality indices and obesity (and its consequent metabolic syndrome) is difficult due to potential confounders, including socio-demographic variables [16]. Among the socio-demographic variables, age, socio-economic and marital statuses are shown to be as predisposing factors of obesity and its-related health problems [17, 18]. Beside lifestyle, psychological factors have also indicated to play a main role in development of obesity and its-related health outcomes by promoting different unhealthy behaviors such as poorer dietary quality and physical inactivity [19, 20]. However, due to unmeasured inter-relationships and high colinearity that exist between psychological factors and other behavioral and lifestyle parameters, direct and indirect mechanisms underlying the association between psychological factors and obesity and its-related health problems have not been well understood. So, according aforementioned, study of complex pathways (including interrelated factors) instead of only assessing direct relationships could help a better understanding and stronger estimate of the role of these variables in development of health outcomes. Structural equation modeling is a relatively novel technique analyzing conceptual models by quantifying the relationships and interactions among a network of factors [21, 22]. The advantage of SEM is the simultaneous assessment of all related pathways considering the role of independent and/or dependent (i.e., mediator) factors in outcome development [22]. To the best of our knowledge, no previous study has evaluated direct and indirect associations between modifiable risk factors and MetS and cardio-metabolic risk factors simultaneously. Therefore, the current study was designed to investigate the associations of HEI-2015 with socio-demographic factors, MetS and other cardio-metabolic risk factors among obese adults.

## Methods

### Participants’ characteristics

This cross-sectional study was conducted among 188 healthy obese adults (96 males and 92 females) aged 20–50 years in Tabriz, Iran. Eligible individuals were recruited using convenience sampling through announcements that provided general information about inclusion criteria (age 20 to 50 years, good health and obesity (BMI≥30)) and placed in hospitals and other public places. A total of 250 participants were willing to participate in the study. After application of the exclusion criteria, 62 individuals (40 women and 22men) were excluded. The exclusion criteria were as follows: pregnancy, lactation, menopause, a history of cardiovascular disease, cancer, Type 2 diabetes mellitus, renal disease, or taking any medications effective for weight loss such as loop diuretics or cortico-steroids, or antidepressants. On the first visit, the aim of the study was described for eligible participants and had them time to discuss question with research coordinator. Written informed consent was obtained from all participants prior to participation in the study. With maximum RMSEA of 0.1 [23], α = 0.05 and power of 80%, a minimum sample size (n) = 184 was calculated using statistica software, version 10. In total, a sample of 188 subjects who agreed to participate was examined in present study. The study protocol was approved by the Ethical Committee of the Tabriz University of Medical Sciences (registration code IR.TBZMED.REC.1396.768). MetS was defined according to the National Cholesterol Education Program (NCEP) Adult Treatment Panel (ATP) III criteria [24]. The presence of at least three of the following risk factors was considered to be MetS: waist circumference > 102 cm or 88 cm (women), blood pressure ⩾ 130/85 mmHg, fasting triglyceride (TG) level ⩾ 150 mg/dl, fasting high-density lipoprotein (HDL) cholesterol level less than 40 mg/dl [7] or 50 mg/dl (women), and fasting blood sugar ⩾ 100 mg/dl.

### Dietary assessment and HEI-2015 calculation

All of information was collected through face to face interview by trained doctoral level nutrition student. A 147-item semi-quantitative food frequency questionnaire (FFQ), with prior evidence of validity and reliability, was used to evaluate usual dietary intake [25, 26]. Trained interviewer asked to participants to report the frequency and amount of each food item consumed during the previous year on a daily, weekly, or monthly basis. The portion sizes of reported foods were converted to grams using household measures. The Iranian Food Composition Table (FCT) was used to analyze nutrient consumption [27]. The USDA FCT was also applied to provide information missing from the Iranian FCT [28]. HEI-2015, the latest version of HEI, reflects the 2015–2020 DGA as an energy-adjusted measure of nutrients (i.e., scores per 1000 calories) [10]. This index consists of nine adequacy and four moderation components with a maximum score of 100. Six adequacy components include total fruits (fruit, fruit juice and canned fruit), whole fruits (fruits except fruit juice), total vegetables, seafood and plant proteins, greens and beans, and total protein foods; each scored at 5 for the highest and 0 for the lowest consumption. A maximum of 10 points was given to other adequacy components (whole grains, dairy and fatty acids). The highest and lowest consumption of four moderation components includes refined grains, sodium, added sugars, and saturated fats received a score of 10 and 0, respectively. Intermediate intakes were scored proportionally. Higher scores in all components indicate a more healthful diet and greater adherence to DGA recommendations.

### Demographic, anthropometric and physical activity assessments

Socioeconomic status (SES) was investigated by gathering information about occupation, educational status, family size and home ownership as individual indicators. The total score was then categorized into three classes of low, middle, and high according to SES tertiles. Physical activity was assessed using the short form of the self-administered International Physical Activity Questionnaire [29].

Weight was measured while participants were minimally clothed without shoes using a Seca scale (Seca, Germany) to the nearest 100 g. Height was also measured in a standing position without shoes using a tape measure with a precision of 0.1 cm. Body mass index (BMI) was computed as weight (kg) divided by square of the height (m^2^). Body composition measurements (fat mass and fat free mass) were performed by bioelectrical impedance analysis (BIA) technology (Tanita, BC-418 MA, Tokyo, Japan). Waist and hip circumference were measured at the narrowest and largest parts, respectively, using a stretch-resistant tape measure with a precision of 0.1 cm over light clothing without any pressure to the body. Waist-to-hip ratio (WHR) was calculated as waist measurement divided by hip measurement. Blood pressure was measured twice after 15 minutes rest in a sitting position using a standardized mercury sphygmomanometer. The average of the two measurements was recorded as the blood pressure.

### Assessment of the mental health

The Depression, Anxiety and Stress Scale 21 (DASS-21), with prior evidence of validity and reliability, was used for the mental health assessment component [30]. Cronbach’s alpha for the DASS questionnaire in Iranian subjects has been reported as 0.77, 0.79 and 0.78 for depression, anxiety and stress, respectively [31]. This questionnaire consists of 7 items for each category of mental health including depression, anxiety, and stress. The responses are rated on a 4-point Likert scale, ranging from zero (“did not apply to me at all”) to 3 (“applied to me very much or most of the time”). An overall score for each scale was calculated by summing the scores for the relevant items and multiplying them by 2 with a range of 0 to 42. Participants were classified into 5 categories: normal, mild, moderate, severe and extremely severe depression, anxiety and stress. Cut-off scores which proposed by Lovibond and Lovibond was used for assigning the severity of each sub-scale [32]. Such that scores ≥ 21, 15 and 26 (for the depression, anxiety and stress, respectively) were labeled as severe [32, 33]. The greater score of each scale showed more severity of mental disorder.

### Appetite measurements

The Visual Analog Scale (VAS) questionnaire was used for appetite measurement. This validated questionnaire includes questions about feelings of hunger, satiation, fullness, prospective food consumption, thirst, and the desire to eat something sweet, salty, or fat [34]. Subjects were asked to make a mark on a 100 mm horizontal line for each question. Scoring was based on measuring the distance from the left side of the line to the mark.

### Biochemical assessments

Blood samples (10 mL) were drawn from all subjects in the morning after 12 hours of overnight fasting. These samples were collected in tubes containing EDTA and serums were immediately separated and frozen at −70°C until assay. Serum glucose, triglyceride (TG), total cholesterol (TC) and high-density lipoprotein cholesterol (HDL-C) were measured by a commercial kit (Pars Azmoon, Tehran, Iran). Serum insulin was also analyzed using the enzyme-linked immunosorbent assay method according to the manufacturer’s instructions (Bioassay Technology Laboratory, Shanghai Korean Biotech, Shanghai City, China). Serum low-density lipoprotein cholesterol (LDL) was calculated according to the Friedewald equation.

### Statistical analysis

The normality of distribution was checked by descriptive measures such as coefficients of skewness and kurtosis, mean and standard deviation [35]. All continuous variables except HOMA-IR and insulin were normally distributed. The mean±SD for normally distributed continuous variables, the median (25 and 75 percentiles) for skewed continuous variables, and the frequency (%) for categorical data are reported. Subjects were categorized based on quartile cutoff points of HEI score including ≤63, 64–67.5, 67.51–73 and ≥74 [36]. Continuous variables were compared across quartile categories of HEI by One-way analysis of variance (ANOVA) with Tukey’s post hoc comparisons. Significant differences in the qualitative variables across quartile categories of HEI were reported using a chi-square test.

**Fig 1:**
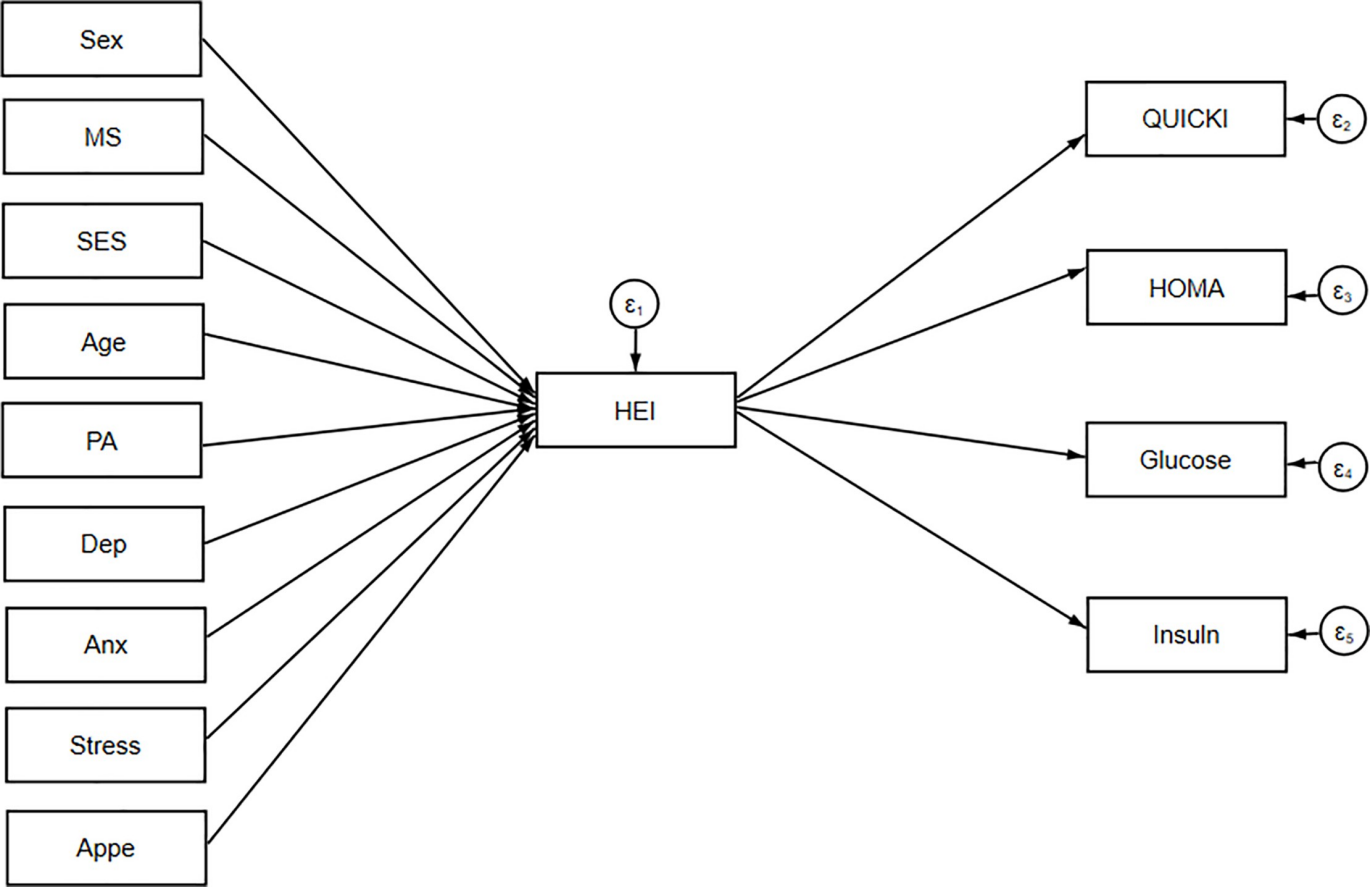
Hypothesized models in which HEI as a mediating variable relates socio-demographic variables and mental health to insulin resistance indices. Abbreviations: HEI, healthy eating index; HOMA, homeostasis model assessment; QUICKI, quantitative insulin sensitivity check index; SES, socio-economic status; PA, Physical activity; MS, marital status; Anx, anxiety; Dep, depression; Appe, appetite.

**Fig 2:**
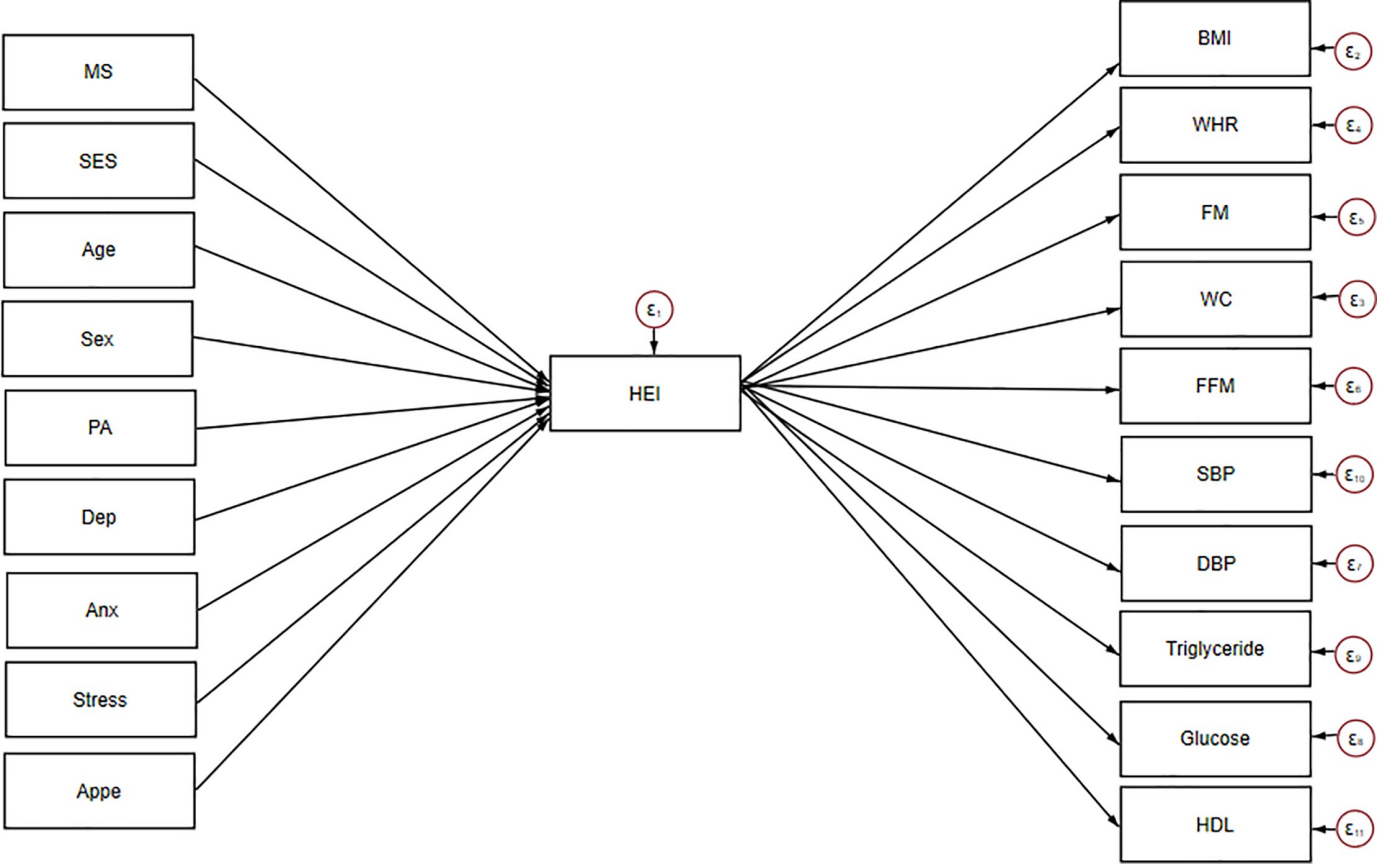
Hypothesized models in which HEI as a mediating variable relates socio-demographic variables and mental health to cardio-metabolic risk factors. Abbreviations: HEI, healthy eating index; WHR, waist–hip ratio; BMI, body mass index; WC, waist circumference; FM, fat mass; FFM, fat free mass; SES, socio-economic status; SBP, systolic blood pressure; DBP, diastolic blood pressure; HDL, high-density lipoprotein; PA, Physical activity; MS, marital status; Anx, anxiety; Dep, depression; Appe, appetite.

**Fig 3:**
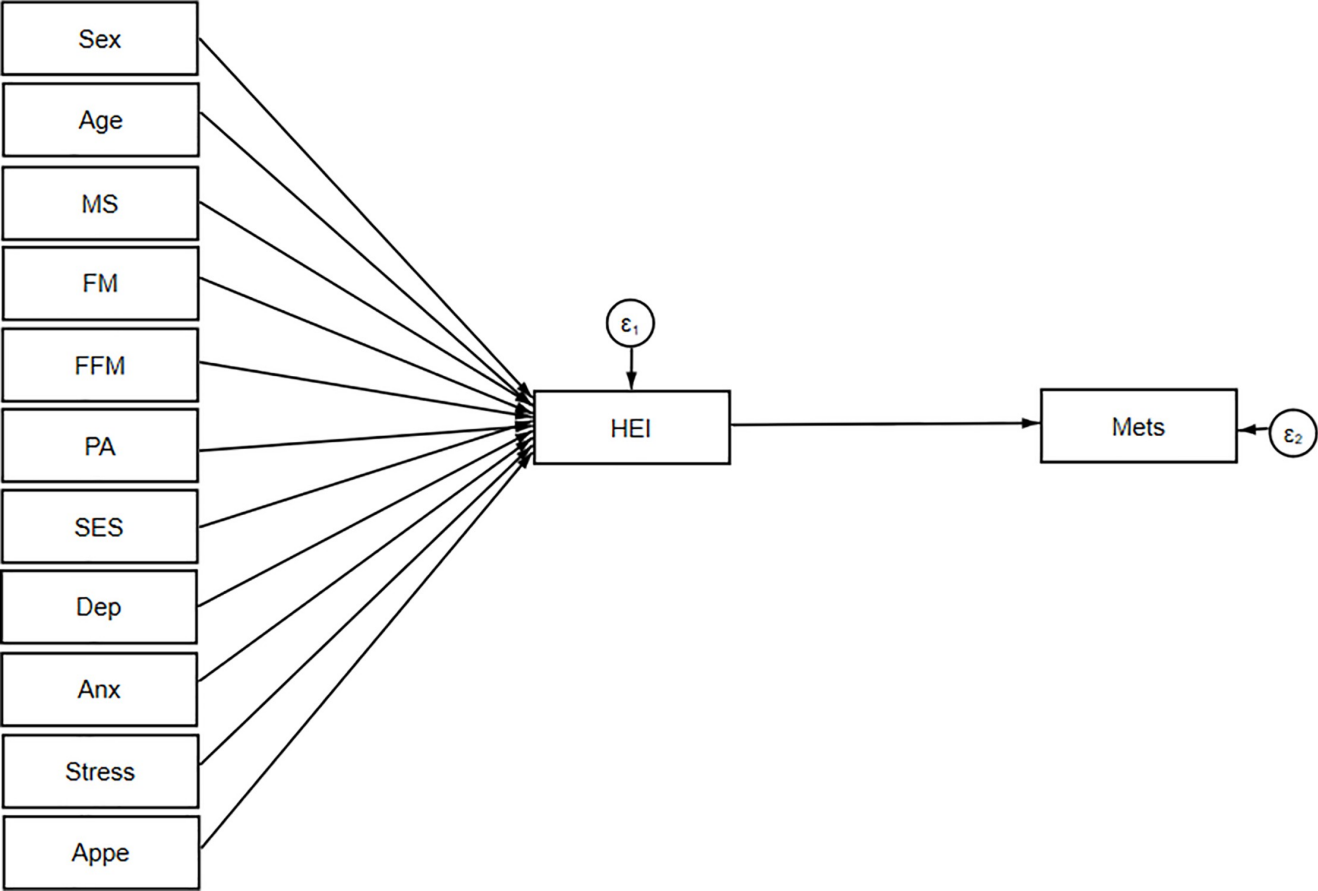
Hypothesized models in which HEI as a mediating variable relates socio-demographic variables and mental health to metabolic syndrome. Abbreviations: HEI, healthy eating index;FM, fat mass; FFM, fat free mass; SES, socio-economic status; MetS, metabolic syndrome; PA, Physical activity; MS, marital status; Anx, anxiety; Dep, depression; Appe, appetite.

SEM was applied to assess the proposed theoretical models. At the first step, conceptual models were developed based on information obtained from literature. Our hypothesized models in which HEI as a mediating variable relates socio-demographic variables and mental health to insulin resistance indices, cardio-metabolic risk factors and metabolic syndrome are summarized in Figs 1, 2 and 3.

Socio-demographic and mental health variables were expected to be directly associated with HEI and on the other hand, HEI was allowed to predict insulin resistance, cardio-metabolic risk factors and Mets in model 1, 2 and 3, respectively. SEM is a combination of two parts, measurement model (latent variables identified using factor analysis), which was not applicable for present study, and the structural model (direct and indirect pathways of associations between latent and other observed variables) [21]. This approach utilizes multiple regression analysis simultaneously within the same analytical framework and models interactions between variables. Several path analyses were run in the current study to test whether: 1) the association between socio-demographic, anthropometric characteristics and insulin resistance indices are mainly mediated by HEI and 2) the association between socio-demographic, mental health and cardio-metabolic risk factors are mediated by HEI. Regression coefficients were estimated using the Maximum Likelihood Estimation Procedure (ML). In the second step, structural equation modeling method was used to explore inter-relations between variables included in models. Modification indices (MIs) were applied to help evaluate and select specific paths for the best-fitting model. In the process of model modification, fit indices were used to help find which of proposed model has the most superior fit. To assess the best fitting model for our data, multiple fit indices were calculated: comparative fit index (CFI) > 0·90 [23], chi-square test (χ2/ degrees of freedom (df) ratio < 5 [37], standardized root mean square residual (SRMR) < 0.08 [38] and root mean square error of approximation (RMSEA) ≤ 0·08 [38]. Totally, in order to get an acceptable model, the sequence of following steps was conducted: initial fit, modification (i.e., using MIs) and refitting. During the model modification process, these steps were repeated until fitting criteria were satisfied. Details of goodness of fit for excluded un-fitted models depicted in Table 1. The significance of indirect effects was evaluated by bootstrap method [39]. The statistical analyses were performed using STATA V.15.0. The final conceptual model (model 3) investigating whether adherence to DGA mediates the effects of demographic and mental health on metabolic syndrome as a binary dependent variable was analyzed in Mplus 8 software. Since this program did not calculate SRMR, this fit index was not included for model 3 (Table 1). A p-value less than 0.05 was considered statistically significant.

**Table 1:** Goodness of fit indices for models.

Model	DF	χ2	χ2 / DF	RMSEA	SRMR	CFI
1^a^	40	670.818	16.77	0.291 (0.272–0.311)	0.134	0.347
1^b^	39	403.256	10.33	0.224 (0.205–0.244)	0.078	0.623
1^c^	33	26.715	0.81	0.000 (0.000–0.038)	0.027	1.000
2^a^	32	1125.435	35.17	0.201 (0.190–0.212)	0.172	0.283
2^b^	131	837.826	6.39	0.170 (0.159–0.181)	0.140	0.490
2^c^	114	118.828	1.04	0.015 (0.000–0.041)	0.055	0.997
3	23	35.273	1.53	0.051 (0.000–0.084)		0.870

χ^2^: Chi-Square value, DF: Degrees of Freedom, RMSEA: Root Mean Square Error of Approximation, SRMR: Standardized Root Mean Square Residual, CFI: Comparative Fit Index.

1^a^, 1^b^ Tested models that did not have an acceptable fit for the association between socio-demographic variables, diet and insulin resistance indices

1^c^ The final model with the best fit according to the values of several fit indices for the association between socio-demographic variables, diet and insulin resistance indices

2^a^, 2^b^ Tested models that did not have an acceptable fit for the association between socio-demographic variables, diet and cardio-metabolic risk factors

2^c^ The final model with the best fit according to the values of several fit indices for the association between socio-demographic variables, diet and cardio-metabolic risk factors

Initial conceptual model 1 was modified as model 1^a^ by adding residual correlations between insulin resistance indices (QUICKI, Insulin and HOMA-IR) to improve model fit and then model 1^a^ was modified as model 1^b^ by adding direct link of age on glucose. On the other hand, initial conceptual model 2 was modified as model 2^a^ by adding residual correlations between body composition indices (BMI, FM, FFM and WC) as well as direct link of sex on WHR. After that, model 2^a^ was modified as model 2^b^ by adding direct link of sex on FFM.

## Results

General characteristics, anthropometric and metabolic parameters of subjects across quartiles of HEI-2015 are presented in Tables 2 and 3, respectively. No significant differences in terms of anthropometric variables and mental health parameters across quartile categories of HEI were reported. A comparison of biochemical parameters between categories of HEI showed that participants in the second quartile of HEI had higher serum concentrations of TC and LDL-C compared with other quartiles. In the present study, the prevalence of MetS was estimated at 33%.

**Table 2:** General characteristics of study participants by quartiles of Healthy Eating Index-2015.

Quartiles of Healthy Eating Index-2015					
	1 (Lowest)	2	3	4 (Highest)	P-value*
**N**	49	45	49	45	
**Gender**					0.606
**Men, n (%)**	25 (26.0)	23 (24.0)	29 (30.2)	19 (19.8)	
**Women, n (%)**	24 (26.1)	22 (23.9)	20 (21.7)	26 (28.3)	
**Age (y)**	36.2 (6.4)	38.4 (7.6)	38.2 (7.6)	39.4 (8.1)	0.195
**Physical activity level, n (%)**					0.637
Low	24 (26.7)	20 (22.2)	19 (21.1)	27 (30)	
Moderate	15 (28.8)	13 (25)	14 (26.9)	10 (19.2)	
High	10 (21.7)	12(26.1)	16 (34.8)	8 (17.4)	
**Marital status, n (%)**					0.790
Married	45 (28.1)	38 (23.8)	40 (25)	37 (23.1)	
Single	4 (15.40	7 (26.9)	9 (34.6)	6 (23.1)	
Divorced	0 (0)	0 (0)	0 (0)	2 (100)	
**Socioeconomic status, n (%)**					0.150
Low	1 (20)	3 (60)	1 (20)	0 (0)	
Middle	31 (31.3)	22 (22.2)	22 (22.2)	24 (24.2)	
High	17 (20.5)	20 (24.1)	26 (31.3)	20 (24.1)	
**Depression, n (%)**					0.576
Normal	35 (25)	33 (23.6)	39 (27.9)	33 (23.6)	
Mild	11 (35.5)	10 (32.3)	6 (19.4)	4 (12.9)	
Moderate	3 (17.6)	2 (11.8)	4 (23.5)	8 (47.1)	
**Anxiety, n (%)**					0.555
Normal	30 (23.1)	32 (24.6)	36 (27.7)	32 (24.6)	
Mild	8 (29.6)	8 (29.6)	6 (22.2)	5 (18.5)	
Moderate	11 (39.3)	5 (17.9)	5 (17.9)	7 (25)	
Severe	0 (0)	0 (0)	1 (50)	1 (50)	
Extremely severe	0 (0)	0 (0)	1 (100)	0 (0)	
**Stress, n (%)**					0.639
Normal	45 (26.8)	40 (23.8)	42 (25)	41 (24.4)	
Mild	3 (20)	4 (26.7)	6 (40)	2 (13.3)	
Moderate	1 (20)	1 (20)	1 (20)	2 (40)	
**Appetite**	33.2 (8.1)	35 (10.2)	34.4 (9.3)	31.7 (8.0)	0.313

*Analysis of variance for continuous variables and χ^2^ test for categorical variables. Data are Mean ±SD.

**Table 3:** Anthropometric and metabolic parameters of study participants by quartiles of Healthy Eating Index-2015.

Quartiles of Healthy Eating Index-2015					
	1 (Lowest)	2	3	4 (Highest)	P-value*
**Weight, kg**	96.4 (12.8)	95.4 (13.7)	97.2(12.3)	95.0 (13.0)	0.847
**Height, m**	166.3 (9.3)	165.2 (9.4)	168.8 (9.3)	165.1 (10.2)	0.202
**BMI (kg/m2)**	34.8 (3.7)	35.0 (3.8)	34.2 (3.9)	34.9 (4.2)	0.736
**FM, (%)**	34.0 (9.8)	33.5 (9.5)	32.8 (9.1)	35.2 (8.1)	0.630
**FFM, (%)**	62.3 (12.7)	62.2 (12.5)	64.5 (12.3)	59.9 (11.9)	0.355
**WC, cm**	109.2 (7.5)	108.8 (11.6)	108.7 (9.8)	108.5 (10.9)	0.987
**WHR**	0.94 (0.07)	0.94 (0.08)	0.94 (0.08)	0.92 (0.07)	0.680
**MetS, n (%)** ^**b**^	16 (25.8)	17 (27.4)	15 (24.2)	14 (22.6)	0.729
**MetS components n (%)**					
Elevated blood pressure (%)	9 (21.4)	10 (23.8)	13 (31)	10 (23.8)	0.543
High serum triacylglycerol %)	11 (26.8)	10 (24.4)	13 (31.7)	7 (17.1)	0.580
Hyperglycemia	13 (31.0)	14 (33.3)	8 (19.0)	7 (16.7)	0.086
Low serum HDL-C (%)	27 (26.7)	23 (22.8)	23 (22.8)	28 (27.7)	0.608
Abdominal adiposity (%)	47 (26.7)	39 (22.2)	47 (26.7)	43 (24.4)	0.693
**LDL, (mg/dl)**	114.7(26.6) ^**c**^	131.7 (35.8)	114.2 (29.5) ^c^	118.1 (29.1)	**0.019**
**HDL, (mg/dl)**	44.76 (8.5)	45.69 (8.4)	45.25 (9.5)	44.18 (9.3)	0.867
**Glucose, (mg/dl)** ^**a**^	92 (86.0, 101)	93 (87.0, 106.5)	88 (83.3, 96)	91 (83.0, 97)	0.247
**Insulin, U/mL**^**a**^	12.1 (8.5, 17.1)	13.9 (9.4, 25.4)	15.9 (10.2, 23.8)	12.0 (8.9, 25.8)	0.396
**HOMA-IR**^**a**^	2.8 (1.8, 4)	3.2 (2.1, 5.7)	3.7 (2.1, 5.3)	2.9 (1.9, 5.5)	0.462
**Total Cholesterol, (mg/dl)**	184.1(29.3) ^d^	201.1 (34.8)	184.7 (35.6) ^e^	184.4 (33.1) ^e^	**0.037**
**SBP (mmHg)**	114.45 (2.51)	116.11 (13.47)	117.35(13.88)	114.27 (15.58)	0.772
**DBP (mmHg)**	74.76 (10.34)	76.89 (11.22)	78.27 (10.92)	75.24 (16.16)	0.485

*Analysis of variance for continuous variables and χ^2^ test for categorical variables. Data are Mean ±SD (all such values) unless indicated.

^**a**^Median (25th and 75th percentile).

WHR, waist–hip ratio; BMI, body mass index; WC, waist circumference; FM, fat mass; FFM, fat free mass; HOMA-IR, homeostasis model assessment of insulin resistance; LDL, low density lipoprotein; HDL, high-density lipoprotein; SBP, systolic blood pressure; DBP, diastolic blood pressure; MetS, metabolic syndrome.

^**b**^Defined as the presence of_3 of the following components: 1) abdominal adiposity (waist circumference > 88 cm); 2) low serum HDL cholesterol (< 50 mg/dL); 3) high serum triacylglycerol (≥150 mg/dL); 4) elevated blood pressure (≥130/85 mm Hg); 5) abnormal glucose homeostasis (fasting plasma glucose ≥110 mg/dL).

^**c**^P <0.05 compared with second quartile, based on Tukey test.

^**d**^p; 0.066 compared with second quartile, based on Tukey test.

^**e**^p: 0.085 compared with second quartile, based on Tukey test

**Fig 4:**
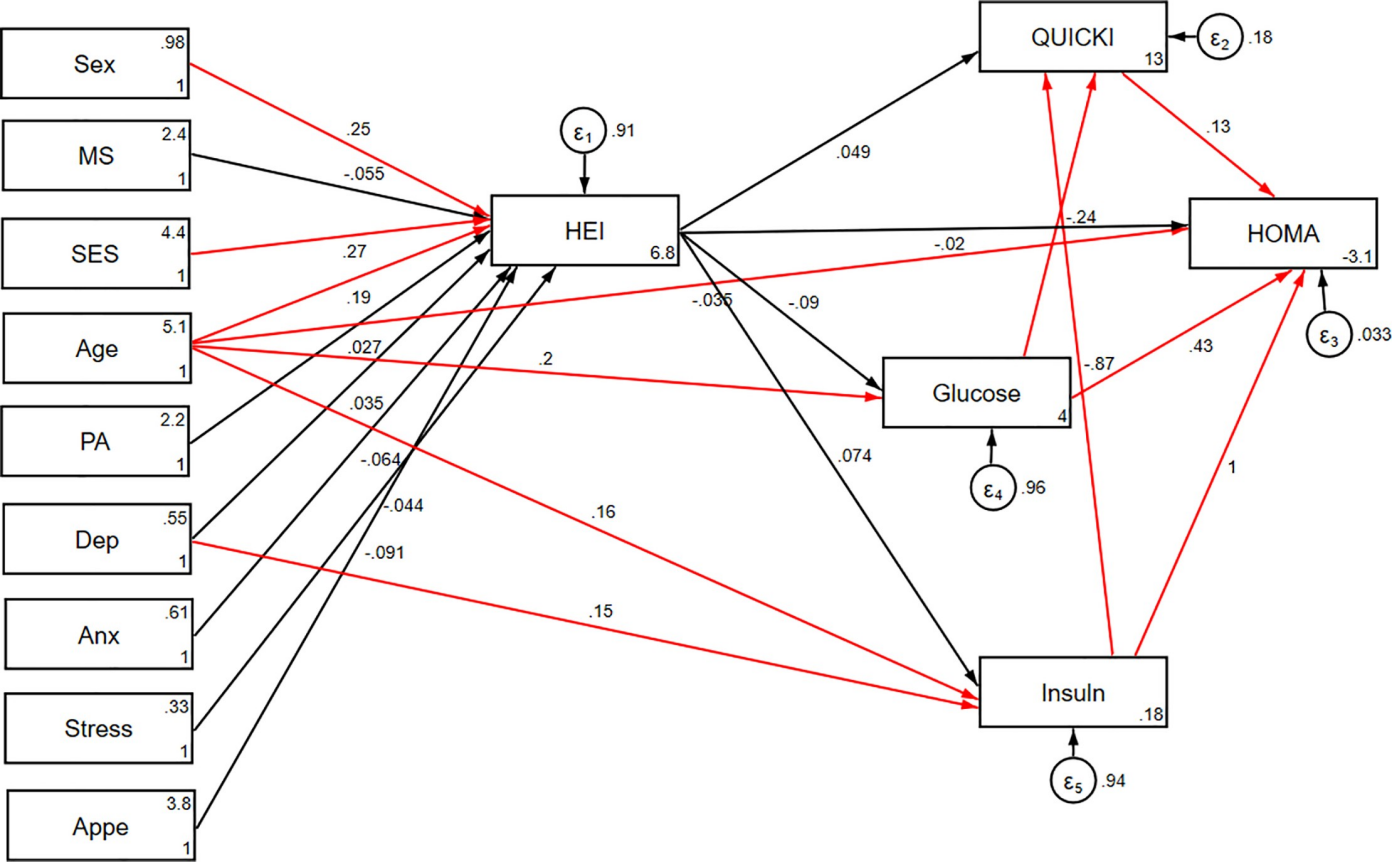
Path analysis diagram with standardized estimates illustrating the total effects of socio-demographic and psychological parameters and diet on insulin resistance indices. Abbreviations: HEI, healthy eating index; HOMA, homeostasis model assessment; QUICKI, quantitative insulin sensitivity check index; SES, socio-economic status; PA, Physical activity; MS, marital status; Anx, anxiety; Dep, depression; Appe, appetite. *All path coefficients are standardized. Red arrows mean p.value ≤ 0.05. ^**£**^Total effect is defined as the sum of direct and indirect effects.

**Fig 5:**
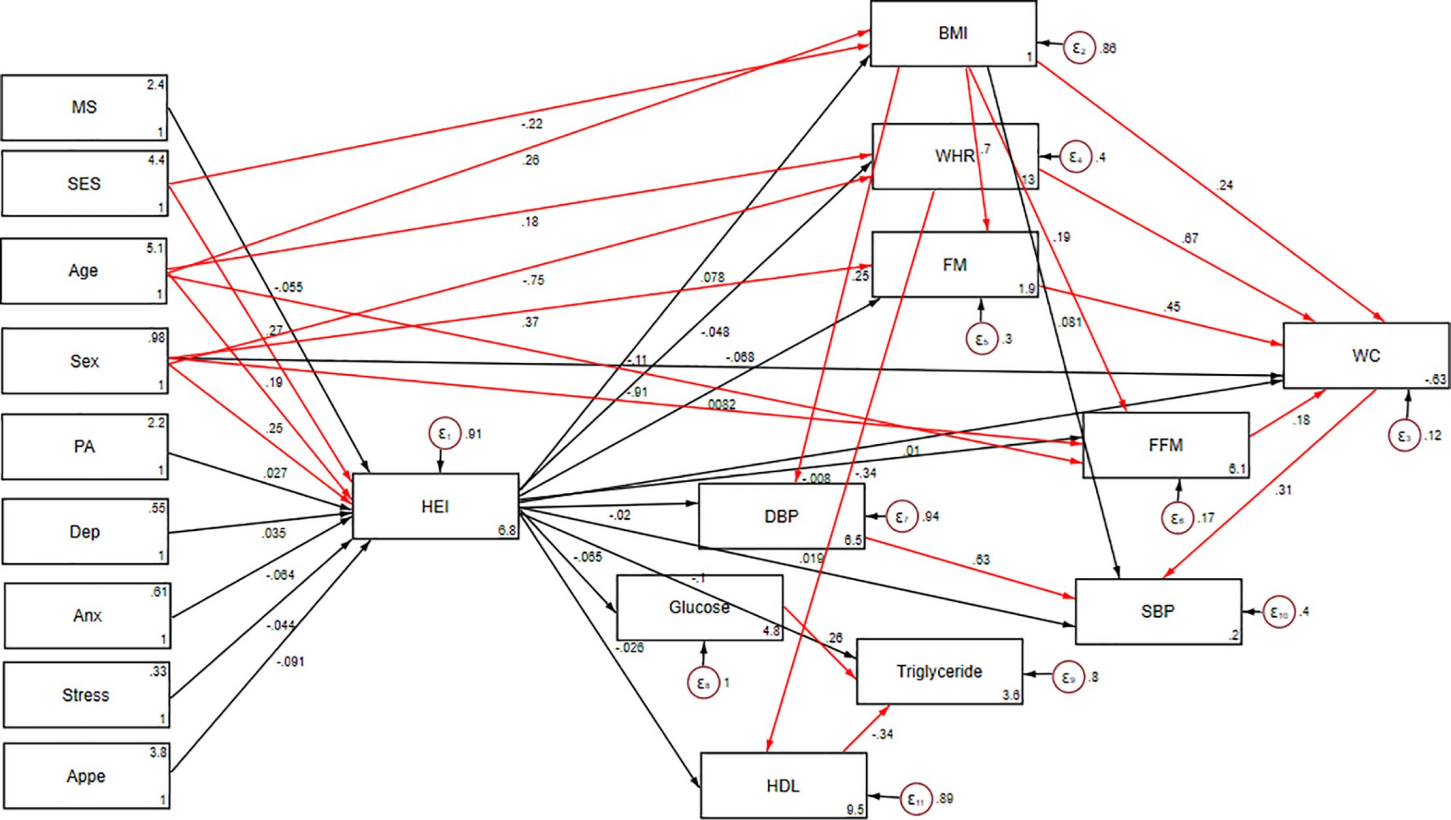
Path analysis diagram with standardized estimates illustrating the total effects of socio-demographic and psychological parameters and diet on cardio-metabolic risk factors. Abbreviations: HEI, healthy eating index; WHR, waist–hip ratio; BMI, body mass index; WC, waist circumference; FM, fat mass; FFM, fat free mass; SES, socio-economic status; SBP, systolic blood pressure; DBP, diastolic blood pressure; HDL, high-density lipoprotein; PA, Physical activity; MS, marital status; Anx, anxiety; Dep, depression; Appe, appetite. *All path coefficients are standardized. Red arrows mean p.value ≤ 0.05. ^**£**^Total effect is defined as the sum of direct and indirect effects.

Significant direct and indirect pathways of the association between socio-demographic and psychological variables and insulin resistance indices among obese individuals are presented in Table 4 (model 1). Among socio- demographic parameters, age was found to be indirectly and positively associated with HOMA-IR through mediatory effects of HEI (B = 0.07; p<0.05, respectively). Additionally, HEI partially mediated the negative association of age and depression with quantitative insulin sensitivity check index (QUICKI) (p<0.05). Significant direct and indirect effects of socio-demographic and psychological parameters through HEI on cardio-metabolic risk factors were also examined (model 2, Table 4). HEI also mediated a positive association between age and FM and FFM (p< 0.05). However, indirect effects of age on HDL and SBP were negative (p< 0.05). Moreover, adherence to DGA seemed to mediate association between gender and WC (B = -9.78), SBP (B = -4.83), TG (B = -13.01) and HDL (B = 4.31). HEI also mediated indirect negative effects of SES on FM (B = -0.56), FFM (B = -0.25), SBP (B = -0.55) and DBP (B = -0.3). Path analysis diagrams with standardized estimates illustrating the total effects of socio-demographic and psychological parameters and diet on insulin resistance indices and cardio-metabolic risk factors are shown in Figs 4 and 5, respectively.

**Table 4:** Statistically significant direct and indirect pathways of the association between socio-demographic and psychological variables and insulin resistance indices and cardio-metabolic risk factors among obese individuals using SEM.

Model Path	Standardized estimate *	SE	P
**Model 1** **Direct effects**			
Gender → HEI	3.887	1.325	0.003
Age → HEI	0.197	0.081	0.015
SES → HEI	3.812	1.194	0.001
Age → HOMA	0.012	0.005	0.012
Age → Glucose	0.592	0.219	0.007
Age → Insulin	0.199	0.089	0.025
Depression → Insulin	2.145	1.025	0.036
**Indirect effects via HEI**			
Age → HOMA	0.076	0.024	0.000
Age → Quicki	-0.001	0.000	0.007
Depression → Quicki	-0.006	0.003	0.050
**Residual covariance**			
Glucose and HOMA	0.048	0.002	0.000
Insulin and HOMA	0.274	0.008	0.000
Quicki and HOMA	0.195	0.006	0.000
Glucose and Quicki	-0.000	0.000	0.000
Insulin and Quicki	-0.003	0.000	0.000
Glucose and HOMA	-0.003	0.001	0.000
Insulin and HOMA	-0.031	0.007	0.000
**Model 2**			
**Direct effects**			
Gender → HEI	3.529	1.302	0.007
Age → HEI	0.189	0.081	0.019
SES → HEI	0.748	0.258	0.004
SES→ BMI	-0.350	0.109	0.001
Age → BMI	0.121	0.037	0.001
Age → WHR	0.002	0.000	0.000
Gender → WHR	-0.113	0.007	0.000
Gender → FM	6.335	0.604	0.000
Age → FFM	-0.201	0.051	0.000
Gender → FFM	-23.280	0.755	0.000
**Indirect effects via HEI**			
Gender → WC	-9.774	1.495	0.000
SES → FM	-0.562	0.197	0.020
Age → FM	0.229	0.065	0.001
SES → FFM	-0.246	0.093	0.009
Age → FFM	0.092	0.031	0.015
SES → DBP	-0.300	0.140	0.039
Gender → TG	-13.010	3.598	0.010
SES → SBP	-0.547	0.222	0.023
Age → SBP	0.275	0.075	0.002
Gender → SBP	-4.825	1.125	0.000
Age → HDL	-0.073	0.028	0.002
Gender → HDL	4.310	0.982	0.001
**Residual covariance**			
BMI and WC	0.101	0.011	0.000
WHR and WC	0.856	0.089	0.000
FM and WC	0.342	0.061	0.000
FFM and WC	0.103	0.047	0.027
BMI and FM	0.176	0.077	0.000
BMI and FFM	0.732	0.101	0.000
BMI and DBP	0.856	0.204	0.000
Glucose and TG	0.684	0.169	0.000
HDL and TG	-0.224	0.043	0.000
WC and SBP	0.430	0.083	0.000
DBP and SBP	0.796	0.062	0.000
WHR and HDL	-0.393	0.012	0.000
BMI and WC	0.680	0.113	0.000

Abbreviations: HEI, healthy eating index; WHR, waist–hip ratio; BMI, body mass index; WC, waist circumference; FM, fat mass; FFM, fat free mass; HOMA, homeostasis model assessment; QUICKI, quantitative insulin sensitivity check index; SES, socio-economic status; SBP, systolic blood pressure; DBP, diastolic blood pressure; TG, triglycerides; LDL, low density lipoprotein; HDL, high-density lipoprotein; MetS, metabolic syndrome, SE; standard error of the estimate

All standardized path coefficients and standardized residual covariance shown were significant (P<0.05).

* Standardized path coefficients and standardized residual covariance coefficients.

**Fig 6:**
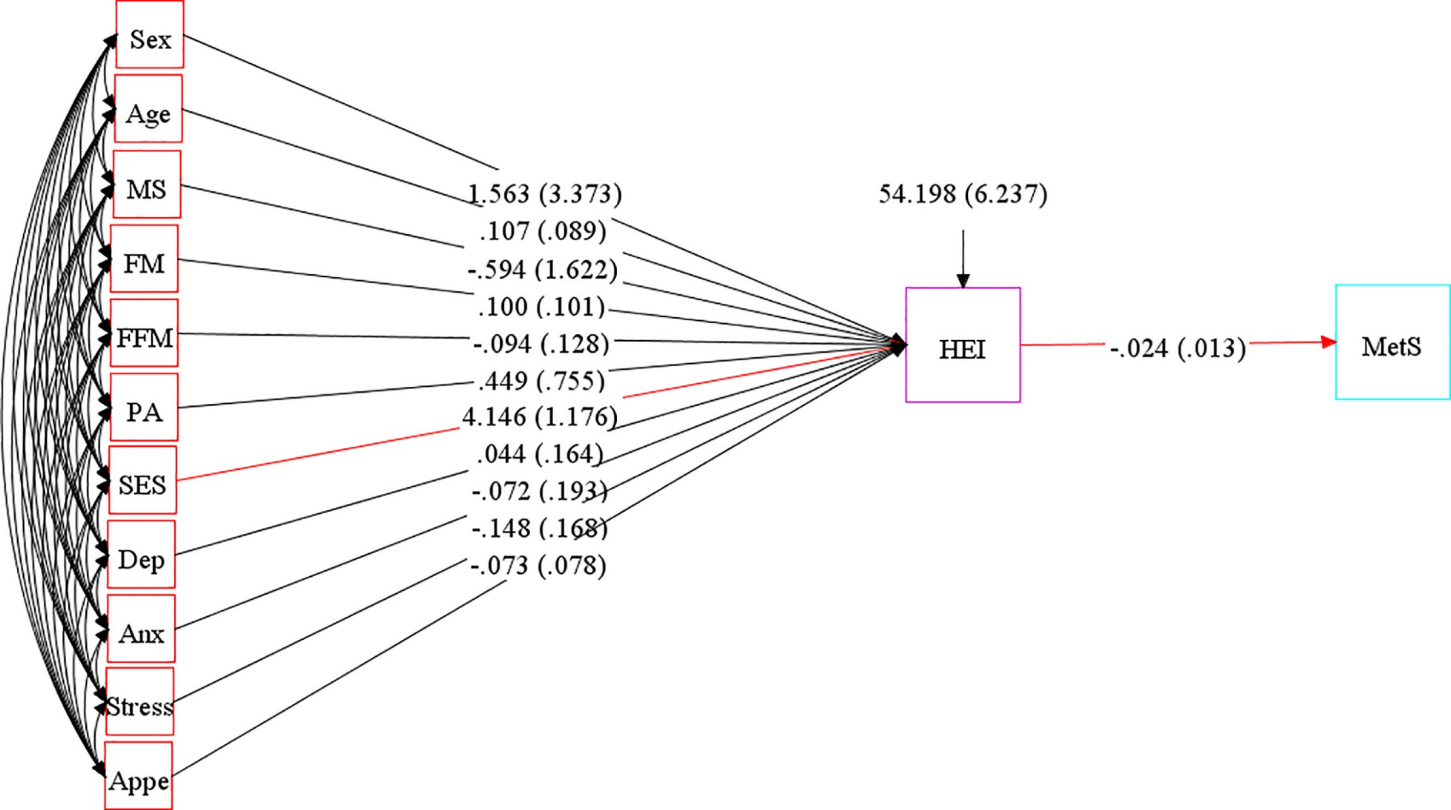
Structural equation model diagram with standardised estimates for total effects of socio-demographic and psychological parameters and diet on Mets. Abbreviations: HEI, healthy eating index;FM, fat mass; FFM, fat free mass; SES, socio-economic status; MetS, metabolic syndrome; PA, Physical activity; MS, marital status; Anx, anxiety; Dep, depression; Appe, appetite. *All path coefficients are standardized. Red arrows mean p.value ≤ 0.05. ^**£**^Total effect is defined as the sum of direct and indirect effects.

Table 1 presents goodness-of-fit indices for SEM models in detail. The final best fitting models had adequate goodness of fit indices (χ^2^/df = 0.81; CFI = 1.00; RMSEA (95% CI) = 0.00 (0.000–0.038); SRMR = 0.027 and χ^2^/df = 1.04; CFI = 0.99; RMSEA (95% CI) = 0.01 (0.000–0.041); SRMR = 0.05 for models 1 and 2, respectively). Significant residual covariances between glycemic indices (model 1) and cardio-metabolic risk factors (model 2) were indicated in Table 4. The results of adjusted final conceptual model (model 3) investigating total effects of socio-demographic and psychological parameters and diet on metabolic syndrome is presented in Fig 6 and Table 5. High adherence to HEI was found to be inversely associated with MetS risk (B = -0.02, *p*<0.05). On the other hand, SES was a significant predictor of HEI, where a greater SES score was related to higher HEI (B = 4.15, *p*<0.05). No association was found between other socio-demographic and mental factors and MetS. The goodness-of-fit indices for the final structural model indicated good fit (χ^2^/df = 1.53; CFI = 0.87; RMSEA (95% CI) = 0.051 (0.000–0.084)).

**Table 5:** Total effects of socio-demographic and psychological parameters and diet on metabolic syndrome among obese individuals using SEM.

	Total ^£^		
Model 3	Standardized estimate	SE	P.value
HEI → MetS	-0.024	0.013	**0.029**
FM → HEI	0.100	0.101	0.160
FFM → HEI	0.094	0.128	0.232
Depression → HEI	0.044	0.164	0.395
Anxiety → HEI	-0.072	0.193	0.354
Stress → HEI	-0.148	0.168	0.189
Gender → HEI	1.563	3.373	0.321
Appetite → HEI	-0.073	0.078	0.175
Age → HEI	0.107	0.089	0.114
SES → HEI	4.146	1.176	**0.000**
Marital status → HEI	-0.594	1.622	0.357
PA → HEI	0.449	0.755	0.276

Abbreviations: HEI, healthy eating index; WHR, waist–hip ratio; BMI, body mass index; WC, waist circumference; FM, fat mass; FFM, fat free mass; SES, socio-economic status; MetS, metabolic syndrome; PA, Physical activity; SE; standard error of the estimate.

^**£**^Total effect is defined as the sum of direct and indirect effects.

## Discussion

To our knowledge, the present study is the first assessment of direct and indirect effects of socio-demographic parameters on MetS and cardio-metabolic risk factors among obese adults using structural equation modeling. Several key findings were revealed in the present work. First, HEI was an independent predictor of MetS, where higher adherence to the healthy eating index was associated with a lower risk of MetS. The association between SES and HEI was positive. Second, examination of the direct and indirect effects of socio-demographic factors on cardio-metabolic risk factors indicated that adherence to ADG mediates the association of age, gender, and SES with cardio-metabolic risk factors. Third, HEI appears to mediate the unfavorable effects of depression and age on some insulin resistance indices.

It has been confirmed that assessment of overall diet quality instead of nutrients or food components is more effective for detecting an association between diet and disease [13]. HEI, as an indicator of diet quality, has been used for identifying nutrition balance and for prediction of health risk [40]. However, there are inconsistencies in risk prediction of disease in diet quality due to unmeasured interrelationships with various effects of modifiers or mediators [12]. Moreover, common statistical methods are unable to exactly calculate the relationship and interrelationship of diet quality and health risk. SEM may be a useful approach to assess this association under a conceptual model by investigating all relevant regression pathways, including direct and indirect, simultaneously [41]. Applying SEM is required for assessment of the mediating role of HEI in the relationship between socio-demographic parameters and MetS and cardiovascular risk factors as well. Additionally, this approach permits a comprehensive view of such an association and allows a more exact interpretation of findings.

Although other observational studies have investigated the association of HEI with MetS [36, 42], the present study is the only SEM modeling study in this regard. Most previous studies, in line with our findings, have reported a negative association between HEI and risk of MetS [36, 42, 43]. However, studies with cohort design are scarce and thus this association has not been completely explored.

Our findings regarding the mediation effect of HEI on the association between age and cardio-metabolic factors are supported by previous studies. A reversal of diet quality-induced obesity [13] and the HDL-C reversion effect of HEI were already confirmed [36]. On the other hand, our results regarding a positive direct association between age and HEI suggests that older people have better diet quality than younger people. This inconsistency suggests that age-related reduction of physical activity may be a cause of age-related increases in obesity indices. This finding has been confirmed in other studies [44]. Koksal et al. reported that older people had higher total scores in diet quality compared to other age groups [44]. Additionally, this study found gender inequalities regarding diet quality and WC, SBP TG and HDL: our results showed women had better dietary guideline compliance [45] and consequently better metabolic status than men. Imamura et al., in a systematic assessment of men and women in 187 countries, reported that women had better dietary patterns compared to men [46]. However, there is a lack of evidence regarding gender differences in the relationship between diet quality and cardio-metabolic risk factors.

It is recognized that SES indicators (e.g., education, occupation, income, etc.) have been inversely associated with chronic diseases through life-style related parameters such as diet [47–49]. In this study, our findings indicate that SES is positively related to HEI. In spite of most previous observational studies showing a positive relationship between SES and other diet quality indices [50, 51], there is no evidence to support independent and specific relationships between SES and HEI. Moreover, in the current study, SES was found to be related to FM, FFM and blood pressure variables through the mediation of HEI. Literature has shown that SES is linked to life style factors such as diet quality, and, in turn, diet quality can contribute to the risk of chronic disease such as obesity [51–53]. Recently Viego et al. documented a negative relationship between SES and prevalence of hypertension, hypercholesterolemia, and diabetes among Argentina's adult population [54]. Likewise, a systematic review of the association between SES and obesity from developed and upper-middle income countries showed that socio-economically disadvantaged adults were more likely to be obese [55]. The causal mechanisms in which SES influences dietary quality have been not yet clearly established. It seems that socio-economically advantaged subjects may have better access to healthy foods, more food security, and higher educational attainment which may affect dietary knowledge and thus a choice of a healthier diet [56]. The present study confirms findings from previous studies regarding a positive association between depression and insulin resistance indices (i.e., insulin, QUICKI. and HOMA) [57, 58]. The results of a systematic review of the literature and a meta-analysis show a small but significant relationship between depression and insulin resistance [59]. On the other hand, findings from several studies have confirmed that adherence to a high-quality diet is associated with lower insulin resistance and hence a lower risk of diabetes development [60, 61]. Therefore, it seems that common mental disorders such as depression can influence insulin resistance through the mediation of HEI. Although underlying mechanisms of observed association are not confirmed, the hypothesis of hypothalamic-pituitary-adrenal (HPA) axis hyperactivity related to depression may explain insulin resistance among depressed people [62]. Enhanced levels of cortisol and other catecholamines which antagonize insulin action on glucose metabolism in depressed people can result in insulin resistance [63]. In addition, elevated appetite with a preference for energy dense foods as a consequence of an increased release of glucocorticoids can contribute to increased insulin resistance among subjects with depressive disorder [64]. The current study has some limitation that must be considered in interpreting these results. First, due to the cross-sectional design of the study, causality cannot be inferred and longitudinal studies are required to infer true Causal Relations. Second, since sample size of present study was relatively small and SEM analyses are highly dependent on the sample size, our results should be interpreted with caution. On the other, our study included only patients with obesity that makes it difficult to generalize our finding to other population, Third, potential biases from under-reporting of dietary intake, especially by obese individuals, may be lead to null results [65]. For this reason, upper and lower extreme values of dietary intake were excluded. Fourth, since other effective factors, for instance meal and snacking patterns and dietary habits, were not considered in this study, the observed associations are not completely explained. Fifth, residual confounding due to unknown or unmeasured confounders in this study cannot be excluded. Last, since dietary intake and other socio-demographic parameters in Tabriz may be different from those in other parts of the country, our results cannot be extended to all Iranians. Regardless of these potential limitations, to our knowledge, this is the first study to examine the mediation role of HEI in the relation between socio-demographic parameters and risk of MetS and cardiovascular risk factors among obese adult using structural equation modeling. Moreover, applying a reliable [26] and validated FFQ [25] to obtain dietary information was an important strength of this study. In conclusion, the findings of the present study suggest that the association between socio-demographic parameters and MetS and cardio-metabolic risk factors in obese adults can be largely explained by diet quality. Moreover, this study shows that psychological depression is related to insulin resistance through the mediation of HEI. A positive association between SES and HEI was found in our model. Further prospective study is needed to confirm the findings of this study.

## Supporting information

S1 Dataset (XLSX)

S1 File Consent form. Persian version.
(DOC)

S2 File Consent form. English version.
(DOCX)

S3 File DASS-21. English version.
(PDF)

S4 File DASS-21. Persian version.
(DOCX)

S5 File Demographic questionaire.English version. (DOCX)

S6 File Demographic questionaire.Persian version. (DOCX)

S7 File FFQ. English version.
(DOCX)

S8 File FFQ. Persian version.
(DOCX)

S9 File IPAQ questionaire. English version.
(PDF)

S10 File IPAQ. Persian version.
(DOCX)

S11 File VAS. English version.
(PDF)

S12 File VAS. Persian version.
(DOCX)
