# Supplementary material for: A structural equation modeling approach for the association of a healthy eating index with metabolic syndrome and cardio-metabolic risk factors among obese individuals
Source: PLoS One. 2019 Jul 1;14(7):e0219193. doi: 10.1371/journal.pone.0219193 (PMC6602284; doi:10.1371/journal.pone.0219193)
Supplement: S1 File — Persian version. (DOC) [file pone.0219193.s002.doc]

**برگ دوم**

**رضايت آگاهانه**

**كد / شماره مطالعاتي:**

**عنوان تحقيق:**

لطفا" علامت گذاري كنيد:

1- من تائيد مي كنم كه برگ اطلاعات مشاركت كننده به تاريخ را براي انجام تحقيق فوق خوانده و فهميده ام و اين فرصت براي من داده شده كه سوالات مورد نظرم را بپرسم.

2- من ميدانم كه شركت من در اين تحقيق داوطلبانه است. من همچنين مي دانم كه من هر زماني كه بخواهم مي توانم از تحقيق كنار بكشم بدون اينكه ملزم به ارائه دليل باشم.

3- من موافقت مي كنم كه در مطالعه/ تحقيق فوق شركت نمايم.

نام مشاركت كننده تاريخ: امضاء

نام محقق تاريخ: امضاء

رونوشت:

- مشاركت كننده
- محقق
