# Supplementary material for: A structural equation modeling approach for the association of a healthy eating index with metabolic syndrome and cardio-metabolic risk factors among obese individuals
Source: PLoS One. 2019 Jul 1;14(7):e0219193. doi: 10.1371/journal.pone.0219193 (PMC6602284; doi:10.1371/journal.pone.0219193)
Supplement: S2 File — English version. (DOCX) [file pone.0219193.s003.docx]

**Project ID number:**

# Project Title:

**Principal Investigator(s):**

I acknowledge that

- 1. I have read what this study is about and understood the risks and benefits. I have had adequate time to think about this and had the opportunity to ask questions and my questions have been answered.
  2. My participation is voluntary and that I am free to withdraw from the project at any time without explanation
  3. I agree to participate in this project.

**Name of Participant:** ……………………………………………………………………………

**Signature & Date:** ……………………………………………………………

**Name of investigator:** ……………………………………………………………….

**Signature & Date:** ……………………………………………………………
