# Supplementary material for: A structural equation modeling approach for the association of a healthy eating index with metabolic syndrome and cardio-metabolic risk factors among obese individuals
Source: PLoS One. 2019 Jul 1;14(7):e0219193. doi: 10.1371/journal.pone.0219193 (PMC6602284; doi:10.1371/journal.pone.0219193)
Supplement: S4 File — Persian version. (DOCX) [file pone.0219193.s005.docx]

**بسمه تعالی**

**جنس: پسر دختر سن: ......... وضعیت تاهل: مجرد متاهل**

**سطح تحصیلات: .......................... رشته: ................. شغل:.........**

**با سلام، فهرست زیر در مورد بخشی از مسائل و مشکلاتی است که ممکن است شما با آن روبرو شده باشید. لطفاً هر یک از عبارات زیر را به دقت بخوانید و پاسخ دهید که این مشکل از هفته گذشته تا به حال چه اندازه برای شما رخ داده است. خواهشمند است پاسخ های خود را با علامت ضربدر (x) مشخص کنید.**

| **ردیف** | **عبارات** | **هیچ وقت** | **کمی** | **گاهی** | **همیشه** |
| --- | --- | --- | --- | --- | --- |
| **1** | **برایم مشکل است که آرام بگیرم.** |  |  |  |  |
| **2** | **متوجه شده ام که دهانم خشک می شود.** |  |  |  |  |
| **3** | **فکر نمی کنم بتوانم هیچ نوع احساس خوبی(احساس مثبت) را تجربه کنم.** |  |  |  |  |
| **4** | **تنفس کردن برایم مشکل است.** |  |  |  |  |
| **5** | **برایم سخت است که در انجام کار پیش قدم شوم.** |  |  |  |  |
| **6** | **به موقعیت هایم به طور افراطی واکنش نشان می دهم.** |  |  |  |  |
| **7** | **در بدنم احساس لرزش می کنم (مثلاً در دست ها و پاها).** |  |  |  |  |
| **8** | **احساس می کنم انرژی روانی بسیاری مصرف می کنم.** |  |  |  |  |
| **9** | **نگرانم که مبادا در بعضی موقعیت ها دچار ترس شوم یا به کار احمقانه ای دست بزنم.** |  |  |  |  |
| **10** | **احساس می کنم چیزی ندارم که منتظرش باشم.** |  |  |  |  |
| **11** | **خودم را پریشان و سردرگم احساس می کنم.** |  |  |  |  |
| **12** | **آرام بودن و در آرامش به سر بردن برایم مشکل است.** |  |  |  |  |
| **13** | **احساس دل مردگی و دل شکستگی دارم.** |  |  |  |  |
| **14** | **نسبت به هر چیزی که مرا از کار باز دارد بی تحکل و نابردبارم.** |  |  |  |  |
| **15** | **احساس می کنم که هر لحظه ممکن است دچار ترس و وحشت شوم.** |  |  |  |  |
| **16** | **قادر نیستم درباره خیلی چیزها شور و اشتیاقی از خود نشان دهم.** |  |  |  |  |
| **17** | **احساس می کنم به عنوان یک فرد ارزش زیادی ندارم.** |  |  |  |  |
| **18** | **فکر می کنم بسیار حساس و زودرنج هستم.** |  |  |  |  |
| **19** | **بدون این که هیچگونه فعالیت بدنی انجام دهم، متوجه شده ام که قلبم غیر عادی کار می کند. (مثلاً ضربان شدید قلب یا از کار افتادن آن برای چند لحظه)** |  |  |  |  |
| **20** | **بدون هیچ دلیل موجهی احساس ترس می کنم.** |  |  |  |  |
| **21** | **احساس می کنم زندگی بی معنا است.** |  |  |  |  |

**با تشکر – پیروز و موفق باشید**
