# Supplementary material for: A structural equation modeling approach for the association of a healthy eating index with metabolic syndrome and cardio-metabolic risk factors among obese individuals
Source: PLoS One. 2019 Jul 1;14(7):e0219193. doi: 10.1371/journal.pone.0219193 (PMC6602284; doi:10.1371/journal.pone.0219193)
Supplement: S5 File — (DOCX) [file pone.0219193.s006.docx]

Please answer all of the following questions as honestly as possible. It is worth noting that the questionnaire information is completely confidential.

**Personal Information**

| What’s your marital status?  Single□ Married□ Widowed□ Divorced□ | Please include your gender:  Male□ Female□  Age: | Please include your name? | ID: |
| --- | --- | --- | --- |
| How is your house ownership?  Own house □ rent house□ | Haw many do you live with in your household?  1 □ 2 □ 3 □ 4 □ 5 □ 6 □ >6 □ | What is the highest level of education you have completed?  Illiterate□ elementary□ middle□diploma□associate degree□ Bachelors□ Master’s and higher□ | Whats your current occupation? |
| Medication you take at the present: | | Do you have any history of following diseases?  Hyperlipidemia□ hypertension□ cancer□ cardiovascular diseases□ diabetes□ renal diseases□  Other diseases: | |

**Phone number**:
