# Supplementary material for: A structural equation modeling approach for the association of a healthy eating index with metabolic syndrome and cardio-metabolic risk factors among obese individuals
Source: PLoS One. 2019 Jul 1;14(7):e0219193. doi: 10.1371/journal.pone.0219193 (PMC6602284; doi:10.1371/journal.pone.0219193)
Supplement: S6 File — (DOCX) [file pone.0219193.s007.docx]

**بسمه تعالی**

**فرم ثبت اطلاعات مراجعه کنندگان**

خواهشمند است برای هر چه دقیق‌تر شدن نتایج این پژوهش موارد زیر را با دقت تکمیل فرمایید. شایان ذکر است که اطلاعات پرسشنامه کاملاً محرمانه می‌باشد.

**مشخصات فردی**

| شماره پرونده: | نام و نام خانوادگی: | سن:  جنس:  مرد□ زن□ | وضعیت تاهل:  مجرد□ متاهل□ مطلقه□ همسرفوت شده□ |
| --- | --- | --- | --- |
| شغل: | **تحصیلات:**  بیسواد□ ابتدایی□ راهنمایی□ دیپلم□ فوق دیپلم□ لیسانس□ فوق لیسانس یا بالاتر□ | **در حال حاضر با چند نفر دیگر در یک خانه زندگی میکنید؟**  1□ 2□ 3□ 4□ 5□ 6□ بیشتر از 6□ | **نوع مالکیت منزل مسکونی:**  شخصی□ اجاره ای□ |
| ابتلا به بیماری:  چربی خون بالا□ فشار خون بالا□ سرطان□ ابتلاء به بیماری های قلبی – عروقی، نارسایی کلیوی و دیابت□  سایر بیماری ها : | | **نوع داروهای مصرفی:** | |

**شماره تماس:**
