# Supplementary material for: A structural equation modeling approach for the association of a healthy eating index with metabolic syndrome and cardio-metabolic risk factors among obese individuals
Source: PLoS One. 2019 Jul 1;14(7):e0219193. doi: 10.1371/journal.pone.0219193 (PMC6602284; doi:10.1371/journal.pone.0219193)
Supplement: S7 File — English version. (DOCX) [file pone.0219193.s008.docx]

**Food Frequency Questionnaire**

| **Your name:** | | | **How often do you intake during last year?** | | | | | **ID:** |
| --- | --- | --- | --- | --- | --- | --- | --- | --- |
| **ID** | **Food items** | **Medium serving** | **Day** | **Week** | | **Month** | **Year** | **Details** |
| 1 | Lavash bread | 1 |  |  |  | |  |  |
| 2 | Barbari bread | 1 |  |  |  | |  |  |
| 3 | Sangak bread | 1 |  |  |  | |  |  |
| 4 | Tafton bread | 1 |  |  |  | |  |  |
| 5 | baguette bread | 1, small |  |  |  | |  |  |
| 6 | Cooked rice | 1 T |  |  |  | |  |  |
| 7 | Cooked pasta | ½ C |  |  |  | |  |  |
| 8 | Potatoes | 1, medium |  |  |  | |  |  |
| 9 | French fries | 1 slice |  |  |  | |  |  |
| 10 | Cooked vermicelli | 1 C |  |  |  | |  |  |
| 11 | Cooked thin noodles | 1 C |  |  |  | |  |  |
| 12 | Sweet biscuit (eg. Digestive) | 1 |  |  |  | |  |  |
| 13 | Crachers | 1 |  |  |  | |  |  |
| 14 | cakes | 1 slice |  |  |  | |  |  |
| 15 | Maize | 1, medium |  |  |  | |  |  |
| 16 | Cooked barley | 1 small cup |  |  |  | |  |  |
| 17 | Lentil | 1 small cup |  |  |  | |  |  |
| 18 | Beans | 1 small cup |  |  |  | |  |  |
| 19 | pea Chick | 1 small cup |  |  |  | |  |  |
| 20 | Cooked fava | 1 small cup |  |  |  | |  |  |
| 21 | soya | 1 small cup |  |  |  | |  |  |
| 22 | Small brown lentil (mash) | 1 small cup |  |  |  | |  |  |
| 23 | Yellow split pea | 1 small cup |  |  |  | |  |  |
| 24 | Beef | 1 oz |  |  |  | |  |  |
| 25 | Lamb | 1 oz |  |  |  | |  |  |
| 26 | Ground beef | I T |  |  |  | |  |  |
| 27 | Poultry Chicken | 1 slice, medium |  |  |  | |  |  |
| 28 | \| All types of fish \| \| --- \| | 1 slice, medium |  |  |  | |  |  |
| 29 | Canned fish | ½ can |  |  |  | |  |  |
| 30 | Heart, liver and kidney | 1 skewer |  |  |  | |  |  |
| 31 | Hamburger | 1 |  |  |  | |  |  |
| 32 | Sausage | 1 slice |  |  |  | |  |  |
| 33 | Cocktail sausages | 1 |  |  |  | |  |  |
| 34 | Egg | 1 |  |  |  | |  |  |
| 35 | Intestine, and viscera | 1 slice |  |  |  | |  |  |
| 36 | Tongue | 1 |  |  |  | |  |  |
| 37 | Brain | 1 |  |  |  | |  |  |
| 38 | Sheeps head | 1 slice |  |  |  | |  |  |
| 39 | Sheeps legs | 1 |  |  |  | |  |  |
| 40 | Pizza | 1 |  |  |  | |  |  |
| 41 | Low fat milk (< 2% fat) | 1 C |  |  |  | |  |  |
| 42 | High fat milk (> 2% fat) | 1 C |  |  |  | |  |  |
| 43 | Cocoa milk | 1 C |  |  |  | |  |  |
| 44 | Strained yogurt | 1 T |  |  |  | |  |  |
| 45 | Yogurt, plaine | 1 bowl |  |  |  | |  |  |
| 46 | High fat yogurt | 1 bowl |  |  |  | |  |  |
| 47 | Cheese | 1 oz |  |  |  | |  |  |
| 48 | Cream cheese | 1 oz |  |  |  | |  |  |
| 49 | Dough | 1 C |  |  |  | |  |  |
| 50 | Cream | 1 T |  |  |  | |  |  |
| 51 | Ice cream, regular | ½ C |  |  |  | |  |  |
| 52 | Ice cream | 1 |  |  |  | |  |  |
| 53 | Butter | 1 oz |  |  |  | |  |  |
| 54 | Margarine | 1 oz |  |  |  | |  |  |
| 55 | Kashk (curd) | 1 T |  |  |  | |  |  |
| 56 | Crushed lettuce | 1 C |  |  |  | |  |  |
| 57 | Tomatoe | 1, medium |  |  |  | |  |  |
| 58 | Cucumber | 1, medium |  |  |  | |  |  |
| 59 | Green leafy vegetables | 1 small plate |  |  |  | |  |  |
| 60 | Cooked vegetables | 1 C |  |  |  | |  |  |
| 61 | Pumpkin | 1 slice |  |  |  | |  |  |
| 62 | Summer squash | 1, medium |  |  |  | |  |  |
| 63 | Cooked eggplant | 1, medium |  |  |  | |  |  |
| 64 | Cooked celery | 1 small cup |  |  |  | |  |  |
| 65 | Cooked green peas, | 1 small cup |  |  |  | |  |  |
| 66 | Cooked green peas | 1 small cup |  |  |  | |  |  |
| 67 | Raw carrot | 1, medium |  |  |  | |  |  |
| 68 | Cooked carrot | 1, medium |  |  |  | |  |  |
| 69 | Garlic | 1 clove |  |  |  | |  |  |
| 70 | Raw onion | 1, small |  |  |  | |  |  |
| 71 | Fried onion | 1 T |  |  |  | |  |  |
| 72 | kales | 1 bowl |  |  |  | |  |  |
| 73 | Bell pepper | 1, medium |  |  |  | |  |  |
| 74 | Cooked spinach | 1 C |  |  |  | |  |  |
| 75 | Turnip | 1, medium |  |  |  | |  |  |
| 76 | red and green chili peppers | 1, medium |  |  |  | |  |  |
| 77 | Tomato paste | 1 T |  |  |  | |  |  |
| 78 | Pickles | 1 bowl |  |  |  | |  |  |
| 79 | vegetable, pickled | 1 bowl |  |  |  | |  |  |
| 80 | Cucumbers, pickled | 1, medium |  |  |  | |  |  |
| 81 | Cantaloupe | ¼ C |  |  |  | |  |  |
| 82 | Melons | 1 slice, medium |  |  |  | |  |  |
| 83 | Watermelon | 1 slice, medium |  |  |  | |  |  |
| 84 | Pear | 1, medium |  |  |  | |  |  |
| 85 | Apricot | 1, medium |  |  |  | |  |  |
| 86 | Cherries | 1 small plate |  |  |  | |  |  |
| 87 | Apple | 1, medium |  |  |  | |  |  |
| 88 | Peach | 1, medium |  |  |  | |  |  |
| 89 | nectarine | 1, medium |  |  |  | |  |  |
| 90 | Green plums | 1, medium |  |  |  | |  |  |
| 91 | Fresh fig | 1, medium |  |  |  | |  |  |
| 92 | Dried fig | 1, medium |  |  |  | |  |  |
| 93 | Grapes | 1 medium bunch |  |  |  | |  |  |
| 94 | Kiwi | 1, medium |  |  |  | |  |  |
| 95 | Grapefruit | 1, medium |  |  |  | |  |  |
| 96 | Orange | 1, medium |  |  |  | |  |  |
| 97 | Persimmon | 1, medium |  |  |  | |  |  |
| 98 | Tangerine | 1, medium |  |  |  | |  |  |
| 99 | Pomegranate | 1, medium |  |  |  | |  |  |
| 100 | Dates | 1, medium |  |  |  | |  |  |
| 101 | plums | 1, medium |  |  |  | |  |  |
| 102 | strawberry | 1, medium |  |  |  | |  |  |
| 103 | Banana | 1, medium |  |  |  | |  |  |
| 104 | Sweet lime | 1, medium |  |  |  | |  |  |
| 105 | lemon | 1, medium |  |  |  | |  |  |
| 106 | Orange juice | 1 C |  |  |  | |  |  |
| 107 | apple juice | 1 C |  |  |  | |  |  |
| 108 | Cantaloupe juice | 1 C |  |  |  | |  |  |
| 109 | Raisins | 1 T |  |  |  | |  |  |
| 110 | Berries | 1 small plate |  |  |  | |  |  |
| 111 | Dried berries | 1 |  |  |  | |  |  |
| 112 | Dried fruits | 1 |  |  |  | |  |  |
| 113 | Green olive | 1 |  |  |  | |  |  |
| 114 | Canned fruits | 1 can |  |  |  | |  |  |
| 115 | \| Fruit juice drink \| \| --- \| | 1 |  |  |  | |  |  |
| 116 | Hydrogenated fats | 1 T |  |  |  | |  |  |
| 117 | Oils | 1 T |  |  |  | |  |  |
| 118 | Olive oil | 1 T |  |  |  | |  |  |
| 119 | Animal fat | 1 T |  |  |  | |  |  |
| 120 | Mayonnaise | 1 T |  |  |  | |  |  |
| 121 | Peanut | 1 |  |  |  | |  |  |
| 122 | Almond | 1 |  |  |  | |  |  |
| 123 | Walnut | 1 |  |  |  | |  |  |
| 124 | Pistachio | 1 |  |  |  | |  |  |
| 125 | Hazelnut | 1 |  |  |  | |  |  |
| 126 | Seeds (sunflower,  Squash  ) | 1 bowl |  |  |  | |  |  |
| 127 | noghl | 1 |  |  |  | |  |  |
| 128 | Sugar | 1 tsp |  |  |  | |  |  |
| 129 | Honey | 1 tsp |  |  |  | |  |  |
| 130 | Jam, Syrup | 1 T |  |  |  | |  |  |
| 131 | Soft drinks | 1 C |  |  |  | |  |  |
| 132 | Pastries | 1 |  |  |  | |  |  |
| 133 | Cream pastries | 1, medium |  |  |  | |  |  |
| 134 | Gaz (nougat) | 1 slice |  |  |  | |  |  |
| 135 | Sohan (saffron brittle toffee) | 1 slice |  |  |  | |  |  |
| 136 | puff | 1 pocket |  |  |  | |  |  |
| 137 | chocolate | 1 |  |  |  | |  |  |
| 138 | Tea | 1 C |  |  |  | |  |  |
| 139 | Salt | 1 tps |  |  |  | |  |  |
| 140 | Chips | 1 pocket |  |  |  | |  |  |
| 141 | Coffee | 1, small c |  |  |  | |  |  |
| 142 | Lemon juice | 1 tps |  |  |  | |  |  |
| 143 | Candies | 1 slice, medium |  |  |  | |  |  |
| 144 | Cooked mushroom | ½ C |  |  |  | |  |  |
| 145 | Homemade halvah | 1 T |  |  |  | |  |  |
| 146 | Halvah | 1 T |  |  |  | |  |  |
| 147 | Spices | 1 tsp |  |  |  | |  |  |

**C** = cup, T = tablespoon, **tsp** = teaspoon, oz= ounce
