# Supplementary material for: A structural equation modeling approach for the association of a healthy eating index with metabolic syndrome and cardio-metabolic risk factors among obese individuals
Source: PLoS One. 2019 Jul 1;14(7):e0219193. doi: 10.1371/journal.pone.0219193 (PMC6602284; doi:10.1371/journal.pone.0219193)
Supplement: S8 File — Persian version. (DOCX) [file pone.0219193.s009.docx]

| نام و نام خانوادگي: | | | | | | | | | | | | | | | كد پرسشنامه: ............ | |  |
| --- | --- | --- | --- | --- | --- | --- | --- | --- | --- | --- | --- | --- | --- | --- | --- | --- | --- |
| رديف | | مواد غذايي | | مقدار | | روز | هفته | | ماه | | | | | سال | **ملاحظات** | |  |
| 1 | | نان لواش | | 1 عدد | |  |  | |  | | | | |  |  | |  |
| 2 | | نان بربري | | 1 عدد | |  |  | |  | | | | |  |  | |  |
| 3 | | نان سنگك | | 1 عدد | |  |  | |  | | | | |  |  | |  |
| 4 | | نان تافتون | | 1 عدد | |  |  | |  | | | | |  | ماشيني 🗆 مشهدي🗆 | |  |
| 5 | | نان باگت | | 1 عدد كوچك | |  |  | |  | | | | |  |  | |  |
| 6 | | برنج پخته | | 1بشقاب غذاخوري | |  |  | |  | | | | |  | معمولي🗆 پر🗆 | |  |
| 7 | | ماكاروني پخته | | 1 كفگير | |  |  | |  | | | | |  | سرصاف🗆 پر🗆 | |  |
| 8 | | سيب‌زميني | | 1 عدد متوسط | |  |  | |  | | | | |  |  | |  |
| 9 | | سيب‌زميني سرخ كرده | | 1 خلال | |  |  | |  | | | | |  |  | |  |
| 10 | | ورميشل پخته  (رشته سوپ) | | 1 ليوان | |  |  | |  | | | | |  |  | |  |
| 11 | | رشته آش | | 1 ليوان | |  |  | |  | | | | |  |  | |  |
| 12 | | بيسكويت (با ذكر نوع) | | 1عدد | |  |  | |  | | | | |  | نوع:............... | |  |
| 13 | | كراكر(بيسكويت ترد) | | 1عدد | |  |  | |  | | | | |  |  | |  |
| 14 | | انواع کيکها | | 1 عدد/1 برش متوسط | |  |  | |  | | | | |  | (شامل کيک يزدي، کيک خانگي، کلوچه، تي تاپ و....) | |  |
| 15 | | ذرت و بلال | | 1 عدد متوسط | |  |  | |  | | | | |  |  | |  |
| 16 | | جو يا بلغور پخته | | 1استکان | |  |  | |  | | | | |  |  | |  |
| 17 | | عدس | | 1استکان | |  |  | |  | | | | |  |  | |  |
| 18 | | لوبيا | | 1استکان | |  |  | |  | | | | |  |  | |  |
| 19 | | نخود | | 1استکان | |  |  | |  | | | | |  |  | |  |
| 20 | | باقلا پخته | | 1استکان | |  |  | |  | | | | |  |  | |  |
| 21 | | سويا | | 1استکان | |  |  | |  | | | | |  |  | |  |
| 22 | | ماش | | 1استکان | |  |  | |  | | | | |  |  | |  |
| 23 | | لپه | | 1استکان | |  |  | | | |  | | |  |  | |  |
| 24 | | گوشت گاو يا گوساله | | 1 تكه خورشتي | |  |  | | | |  | | |  |  | |  |
| 25 | | گوشت گوسفند | | 1 تكه خورشتي | |  |  | | | |  | | |  |  | |  |
| 26 | | گوشت چرخ كرده | | 1قاشق غذاخوري | |  |  | | | |  | | |  |  | |  |
| 27 | | مرغ و جوجه | | 1 قطعه متوسط | |  |  | | | |  | | |  | ران🗆 سينه🗆 بال 🗆  با پوست🗆 بي پوست🗆 | |  |
| 28 | | ماهي (بجز تن) با ذكر نوع | | 1 قطعه متوسط (1كف دست كامل) | |  |  | | | |  | | |  | مقدار: ...........  نوع:.............. | |  |
| 29 | | تن ماهي (كنسرو) | | 2/1 قوطي | |  |  | | | |  | | |  | آيا روغن آن دور ريخته مي‌شود؟ بله🗆 خير🗆 | |  |
| 30 | | دل و جگر و قلوه | | 1 سيخ | |  |  | | | |  | | |  |  | |  |
| 31 | | همبرگر | | 1 عدد | |  |  | | | |  | | |  |  | |  |
| 32 | | کالباس | | 1برش | |  |  | | | |  | | |  |  | |  |
| 33 | | سوسيس | | 1عدد(آلماني🗆كوكتل🗆 | |  |  | | | |  | | |  |  | |  |
| **34** | | تخم‌مرغ | | 1 عدد | |  |  | | | |  | | |  |  | |  |
| **35** | | سيرابي و شيردان | | 1 قطعه | |  |  | | | |  | | |  |  | |  |
| **36** | | زبان | | 1 عدد كامل | |  |  | | | |  | | |  |  | |  |
| **37** | | مغز | | 1 عدد كامل | |  |  | | | |  | | |  |  | |  |
| **38** | | كله | | 1 کف دست | |  |  | | | |  | | |  |  | |  |
| **39** | | پاچه | | 1 عدد | |  |  | | | |  | | |  | اگر مقدار و اجزاي خاصي مصرف مي‌شود ذكر شود | |  |
| **40** | | پيتزا | | 1 عدد | |  |  | | | |  | | |  |  | |  |
| **41** | | شير كم‌چرب (کمتر از2%) | | 1 ليوان | |  |  | | | |  | | |  |  | |  |
| **42** | | شير پرچرب (بيشتر يا مساوي2%) | | 1 ليوان | |  |  | | | |  | | |  |  | |  |
| **43** | | شير كاكائو | | 1 ليوان | |  |  | | | |  | | |  |  | |  |
| **44** | | ماست چكيده | | 1 قاشق غذاخوري | |  |  | | | |  | | |  |  | |  |
| **45** | | ماست معمولي | | 1 كاسه ماست‌خوري | |  |  | | | |  | | |  |  | |  |
| **46** | | ماست پرچرب | | 1 كاسه ماست‌خوري | |  |  | | | |  | | |  |  | |  |
| **47** | | پنير | | 1 قوطي كبريت | |  |  | | | |  | | |  |  | |  |
| **48** | | پنير خامه‌اي | | 1 قوطي كبريت | |  |  | | | |  | | |  |  | |  |
| **49** | | دوغ | | 1 ليوان | |  |  | | | |  | | |  |  | |  |
| **50** | | خامه و سرشير | | 1قاشق غذاخوري | |  |  | | | |  | | |  |  | |  |
| **51** | | بستني سنتي | | نصف ليوان | |  |  | | | |  | | |  | چند ماه سال؟ | |  |
| **52** | | بستني غير سنتي | | 1عدد | |  |  | | | |  | | |  | چند ماه سال؟ | |  |
| **53** | | كره | | 1 قوطي كبريت | |  |  | | | |  | | |  |  | |  |
| **54** | | مارگارين | | 1 قوطي كبريت | |  |  | | | |  | | |  |  | |  |
| **55** | | كشك | | 1قاشق غذاخوري | |  |  | | | |  | | |  |  | |  |
| **56** | | كاهو خرد شده | | 1ليوان | |  |  | | | |  | | |  |  | |  |
| **57** | | گوجه‌فرنگي | | 1 عدد متوسط | |  |  | | | |  | | |  |  | |  |
| **58** | | خيار | | 1 عدد متوسط | |  |  | | | |  | | |  |  | |  |
| **59** | | سبزي خوردن | | 1 پيش‌دستي | |  |  | | | |  | | |  |  | |  |
| **60** | | سبزي پخته(آش، پلو، سوپ و غيره) | | 1ليوان | |  |  | | | |  | | |  |  | |  |
| **61** | | كدو حلوايي | | 1کف دست | |  |  | | | |  | | |  |  | |  |
| **62** | | كدو خورشتي | | 1 عدد متوسط | |  |  | | | |  | | |  |  | |  |
| **63** | | بادمجان پخته | | 1 عدد متوسط | |  |  | | | |  | | |  |  | |  |
| **64** | | كرفس پخته | | 1استکان | |  |  | | | |  | | |  |  | |  |
| **65** | | نخود سبز پخته | | 1استکان | |  |  | | | |  | | |  |  | |  |
| **66** | | لوبيا سبز پخته | | 1استکان | |  |  | | | |  | | |  |  | |  |
| **67** | | هويج خام | | 1 عدد متوسط | |  |  | | | |  | | |  |  | |  |
| **68** | | هويج پخته | | 1 عدد متوسط | |  |  | | | |  | | |  |  | |  |
| **69** | | سير | | 1 حبه | |  |  | | | |  | | |  |  | |  |
| **70** | | پياز خام | | 1 عدد کوچک | |  |  | | | |  | | |  |  | |  |
| **71** | | پياز سرخ شده | | 1قاشق غذاخوري | |  |  | | | |  | | |  |  | |  |
| **72** | | انواع كلم | | كاسه ماست‌خوري | |  |  | | | |  | | |  |  | |  |
| **73** | | فلفل دلمه‌اي | | 1 عدد متوسط | |  |  | | | |  | | |  |  | |  |
| **74** | | اسفناج پخته | | 1ليوان | |  |  | | | |  | | |  |  | |  |
| 75 | | شلغم | | 1 عدد متوسط | |  |  | | | |  | | |  |  | |  |
| 76 | | فلفل سبز باريك | | 1 عدد متوسط | |  |  | | | |  | | |  |  | |  |
| 77 | | سس قرمز | | 1قاشق غذاخوري | |  |  | | | |  | | |  |  | |  |
| 78 | | ترشي | | 1پياله | |  |  | | | |  | | |  | چند ماه سال؟ | |  |
| 79 | | شور | | 1پياله | |  |  | | | |  | | |  | چند ماه سال؟ | |  |
| 80 | | خيار شور | | 1 عدد متوسط | |  |  | | | |  | | |  |  | |  |
| 81 | | طالبي و گرمک | | 4/1 عدد | |  |  | | | |  | | |  |  | |  |
| 82 | | خربزه | | 1 قاچ متوسط | |  |  | | | |  | | |  |  | |  |
| 83 | | هندوانه | | 1 قاچ متوسط | |  |  | | | |  | | |  |  | |  |
| 84 | | گلابي | | 1 عدد متوسط | |  |  | | | |  | | |  |  | |  |
| 85 | | زردآلو | | 1 عدد متوسط | |  |  | | | |  | | |  |  | |  |
| 86 | | گيلاس يا آلبالو | | 1 پيش دستي کوچک | |  |  | | | |  | | |  |  | |  |
| 87 | | سيب | | 1 عدد متوسط | |  |  | | | |  | | |  |  | |  |
| 88 | | هلو | | 1 عدد متوسط | |  |  | | |  | | | |  |  | |  |
| 89 | | شليل | | 1 عدد متوسط | |  |  | | |  | | | |  |  | |  |
| 90 | | گوجه سبز | | 1 عدد متوسط | |  |  | | |  | | | |  |  | |  |
| 91 | | انجير تازه | | 1 عدد متوسط | |  |  | | |  | | | |  |  | |  |
| 92 | | انجير خشك | | 1 عدد متوسط | |  |  | | |  | | | |  |  | |  |
| 93 | | انگور | | 1 خوشه متوسط | |  |  | | |  | | | |  |  | |  |
| 94 | | كيوي | | 1 عدد متوسط | |  |  | | |  | | | |  |  | |  |
| 95 | | گريپ فروت | | 1 عدد متوسط | |  |  | | |  | | | |  |  | |  |
| 96 | | پرتقال | | 1 عدد متوسط | |  |  | | |  | | | |  |  | |  |
| 97 | | خرمالو | | 1 عدد متوسط | |  | | |  | | | |  |  | | |  |
| 98 | | نارنگي | | 1 عدد متوسط | |  | | |  | | | |  |  | | |  |
| 99 | | انار | | 1 عدد متوسط | |  | | |  | | | |  |  | | |  |
| 100 | | خرما | | 1 عدد متوسط | |  | | |  | | | |  |  | | |  |
| 101 | | آلو (زرد و قرمز) | | 1 عدد متوسط | |  | | |  | | | |  |  | | |  |
| 102 | | توت فرنگي | | 1عدد | |  | | |  | | | |  |  | | |  |
| 103 | | موز | | 1 عدد متوسط | |  | | |  | | | |  |  | | |  |
| 104 | | ليمو شيرين | | 1 عدد متوسط | |  | | |  | | | |  |  | | |  |
| 105 | | ليمو ترش | | 1 عدد متوسط | |  | | |  | | | |  |  | | |  |
| 106 | | آب پرتغال | | 1 ليوان | |  | | |  | | | |  |  | | |  |
| 107 | | آب سيب | | 1 ليوان | |  | | |  | | | |  |  | | |  |
| 108 | | آب طالبي | | 1 ليوان | |  | | |  | | | |  |  | | |  |
| 109 | | كشمش | | 1قاشق غذاخوري | |  | | |  | | | |  |  | | |  |
| 110 | | توت تازه | | 1 پيش دستي | |  | | |  | | | |  |  | | |  |
| 111 | | توت خشك | | 1عدد | |  | | |  | | | |  |  | | |  |
| 112 | | برگه ها | | 1عدد | |  | | |  | | | |  |  | | |  |
| 113 | | زيتون سبز | | 1عدد | |  | | |  | | | |  |  | | |  |
| 114 | | كمپوت ميوه‌جات | | 1 قوطي | |  | | |  | | | |  |  | | |  |
| 115 | | آبميوه بسته بندي شده | | 1عدد | |  | | |  | | | |  |  | | |  |
| 116 | | روغن نباتي جامد | | 1قاشق غذاخوري | |  | | |  | | | |  |  | | |  |
| 117 | | روغن مايع | | 1قاشق غذا خوري | |  | | |  | | | |  |  | | |  |
| 118 | | روغن زيتون | | 1قاشق غذاخوري | |  | | |  | | | |  |  | | |  |
| 119 | | روغن حيواني | | 1قاشق غذاخوري | |  | | |  | | | |  |  | | |  |
| 120 | | سس مايونز | | 1قاشق غذاخوري | |  | | |  | | | |  |  | | |  |
| 121 | | بادام زميني | | 1عدد | |  | | |  | | | |  |  | | |  |
| 122 | | بادام | | 1عدد | |  | | |  | | | |  |  | | |  |
| 123 | | گردو | | 1 عدد مغز كامل | |  | | |  | | | |  |  | | |  |
| 124 | | پسته | | 1 عدد | |  | | |  | | | |  |  | | |  |
| 125 | | فندق | | 1 عدد | |  | | |  | | | |  |  | | |  |
| 126 | | تخمه (هندوانه كدو,آفتابگردان) | | كاسه ماست خوري | |  | | |  | | | |  |  | | |  |
| 127 | | قند، شكرپنيريا نقل | | 1 عدد | |  | | |  | | | |  |  | | |  |
| 128 | | شكر | | 1قاشق مربا خوري | |  | | |  | | | |  |  | | |  |
| 129 | | عسل | | 1قاشق مربا خوري | |  | | |  | | | |  |  | | |  |
| 130 | | مربا (با نوع) | | 1قاشق غذاخوري | |  | | |  | | | |  |  | | |  |

| 131 | نوشابه هاي کولادار يا صنعتي | 1 ليوان |  |  |  |  |  |
| --- | --- | --- | --- | --- | --- | --- | --- |
| 132 | شيريني خشك | 1 عدد متوسط |  |  |  |  |  |
| 133 | شيريني تر | 1 عدد متوسط |  |  |  |  |  |
| 134 | گز | 1 عدد متوسط |  |  |  |  | آردي 🗆 لقمه‌اي 🗆 |
| 135 | سوهان | 1 قطعه |  |  |  |  |  |
| 136 | پفك | 1 بسته |  |  |  |  |  |
| 137 | شكلات کاکايويي | 1 عدد |  |  |  |  |  |
| 138 | چاي | 1 ليوان |  |  |  |  |  |
| 139 | نمك | 1 قاشق چايخوري |  |  |  |  | نوع:....... |
| 140 | چيپس | 1 بسته |  |  |  |  |  |
| 141 | قهوه | 1 فنجان |  |  |  |  |  |
| 142 | آبليمو | 1 قاشق مربا خوري |  |  |  |  |  |
| 143 | نبات و آبنبات | 1 تكه متوسط |  |  |  |  |  |
| 144 | قارچ پخته | نصف ليوان |  |  |  |  |  |
| 145 | حلوا خانگي | 1قاشق غذاخوري |  |  |  |  |  |
| 146 | حلوا شکري | 1قاشق غذاخوري |  |  |  |  |  |
| 147 | انواع ادويه ها | 1قاشق چاي خوري |  |  |  |  |  |
