# Supplementary material for: A structural equation modeling approach for the association of a healthy eating index with metabolic syndrome and cardio-metabolic risk factors among obese individuals
Source: PLoS One. 2019 Jul 1;14(7):e0219193. doi: 10.1371/journal.pone.0219193 (PMC6602284; doi:10.1371/journal.pone.0219193)
Supplement: S10 File — Persian version. (DOCX) [file pone.0219193.s011.docx]

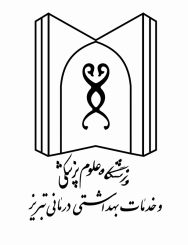


"پرسشنامه بين‌المللي فعاليت بدني"

ما قصد داريم درباره فعاليت‌هاي بدني كه مردم به عنوان بخشي از زندگي روزمره‌شان انجام مي‌دهند، اطلاعات كسب كنيم. سؤالها در مورد زمان‌هايي خواهد بود كه شما در طول **7 روز گذشته** به صورت حركات بدني فعال بوده‌ايد. لطفاً به تك تك سؤالات پاسخ دهيد حتي اگر خود را فرد فعالي به حساب نمي‌آوريد. لطفاً فعاليت‌هايي را كه در محل كار، يا به عنوان بخشي از كار منزل و حياط (باغچه)، رفتن از جايي به جاي ديگر، تمرينات ورزشي و فعاليت‌هايي كه به عنوان سرگرمي در اوقات فراغت انجام مي‌دهيد، مدنظر قرار دهيد.

تمام فعاليت‌هاي **شديدي** را كه در طول **7 روز اخير** انجام داده‌ايد، مدنظر قرار دهيد. فعاليت‌هاي **شديد** به فعاليت‌هايي اطلاق مي‌شود كه قدرت بدني زيادي مي‌خواهد و باعث مي‌شود بسيار شديدتر از حالت عادي نفس بكشيد. لطفاً فقط فعاليت‌هايي را مدنظر قرار دهيد كه **حداقل به مدت 10 دقيقه به صورت پيوسته** انجام داده‌ايد.

1. در طول **7 روز اخير** چند روز آن فعاليت بدني **شديد** مانند بلند كردن اجسام سنگين، حفاري (مثل كندن باغچه)، ايروبيك (ورزش هوازي)، دوچرخه‌‌سواري سريع، فوتبال و دويدن داشته‌ايد؟
   - .............. روز در هفته
   - فعاليت ‌بدني شديد نداشته‌ام □ (مراجعه به سؤال 3)
2. معمولاً چه مدت زماني در چنين روزهايي براي انجام اين فعاليت‌هاي بدني **شديد** به صورت پيوسته صرف كرده‌ايد؟

- ............. ساعت در روز
- ............... دقيقه در روز

فعاليت‌هاي بدني **متوسطي** را كه در طول **7 روز اخير** انجام داده‌ايد، مدنظر قرار دهيد. فعاليت‌هاي فيزيكي **متوسط** به فعاليت‌هايي اطلاق مي‌شود كه قدرت متوسطي مي‌خواهد و باعث مي‌شود شما كمي تندتر از حالت عادي نفس بكشيد.

لطفاً فقط فعاليت‌هايي را مدنظر قرار دهيد كه **حداقل به مدت 10 دقيقه به صورت پيوسته** انجام داده‌ايد.

1. در طول **7 روز اخير** چند روز آن فعاليت فيزيكي **متوسط** مانند حمل بارهاي سبك، دوچرخه‌‌سواري با سرعت متوسط يا واليبال انجام داده‌ايد؟ لطفاً پياده‌روي را به حساب نياوريد.

- .............. روز در هفته
- فعاليت‌ بدني متوسط نداشته‌ام □ (مراجعه به سؤال 5)

1. معمولاً چه مدت زماني در چنين روزهايي براي انجام فعاليت‌هاي بدني **متوسط** صرف كرده‌ايد؟

- ............. ساعت در روز
- .............. دقيقه در روز

لطفاً مدت زماني را كه در طول **7 روزگذشته** به **پياده‌روي** اختصاص داده‌ايد، مدنظر قرار دهيد. اين قسمت پياده روي در محل كار، در خانه، براي رفتن از محلي به محل ديگر و هر نوع پياده روي ديگر كه شما به عنوان تفريح، ورزش، تمرينات جسماني يا در اوقات فراغت انجام داده‌ايد را شامل مي‌شود.

1. در طول **7 روز اخير**، چند روز آن به **مدت حداقل 10 دقيقه و به صورت پيوستهپياده‌روي** داشته‌ايد؟

- .............. روز در هفته
- پياده‌روي نداشته‌ام □ (مراجعه به سؤال 7)

1. معمولاً چه مدت زماني در چنين روزهايي براي **پياده‌روي** صرف كرده ايد؟

- ............. ساعت در روز
- .............. دقيقه در روز
- نمي‌دانم/ مطمئن نيستم

آخرين سؤال مربوط به اوقاتي است كه شما در طول **7 روز اخير** به **نشستن** اختصاص داده‌ايد كه شامل نشستن در محل كار، در خانه، هنگام انجام تكاليف و در اوقات فراغت مي‌باشد. اين زمان نشستن پشت ميز، نشستن يا لم دادن هنگام تماشاي تلوزيون و مطالعه و زماني كه براي نشستن با دوستان و فاميل اختصاص داده‌ايد را هم شامل مي‌شود.

1. در طول **7 روز اخير**، چه مدت زماني را در **هر روز** به **نشستن** اختصاص داده‌ايد؟

الف) ............. ساعت در روز

ب) ............... دقيقه در روز

ج) نمي‌دانم/ مطمئن نيستم

**از همكاري شماسپاسگزاريم**
