# Supplementary material for: A structural equation modeling approach for the association of a healthy eating index with metabolic syndrome and cardio-metabolic risk factors among obese individuals
Source: PLoS One. 2019 Jul 1;14(7):e0219193. doi: 10.1371/journal.pone.0219193 (PMC6602284; doi:10.1371/journal.pone.0219193)
Supplement: S11 File — English version. (PDF) [file pone.0219193.s012.pdf]

Code: **date:** **your name:**

1. **How hungry do you feel?**

**I'm not hungry at all**										**I have never been more hungry**
0	1	2	3	4	5	6	7	8	9	10

2. **How full do you feel?**

**I'm not full at all**										**I'm totally full**
0	1	2	3	4	5	6	7	8	9	10

3. **How strong is your desire to eat?**

**Not at all**										**Very much**
0	1	2	3	4	5	6	7	8	9	10

4. **How strong is your desire to eat something sweet?**

**Not at all**										**Very much**
0	1	2	3	4	5	6	7	8	9	10

5. **How strong is your desire to eat something salty?**

**Not at all**										**Very much**
0	1	2	3	4	5	6	7	8	9	10

6. **How strong is your desire to eat something fat?**

**Not at all**										**Very much**
0	1	2	3	4	5	6	7	8	9	10
