# Supplementary material for: A structural equation modeling approach for the association of a healthy eating index with metabolic syndrome and cardio-metabolic risk factors among obese individuals
Source: PLoS One. 2019 Jul 1;14(7):e0219193. doi: 10.1371/journal.pone.0219193 (PMC6602284; doi:10.1371/journal.pone.0219193)
Supplement: S12 File — Persian version. (DOCX) [file pone.0219193.s013.docx]

**کد: تاریخ: نام و نام خانوادگی:**

1. **تا چه حد احساس گرسنگی می کنید؟**

**اصلا گرسنه نیستم تا بحال اینقدر گرسنه نبوده ام**

|  |  |  |  |  |  |  |  |  |  |
| --- | --- | --- | --- | --- | --- | --- | --- | --- | --- |

**0 1 2 3 4 5 6 7 8 9 10**

1. **تا چه حد احساس سیری می کنید؟**

**اصلا سیر نیستم تا بحال اینقدر سیر نبوده ام**

|  |  |  |  |  |  |  |  |  |  |
| --- | --- | --- | --- | --- | --- | --- | --- | --- | --- |

**0 1 2 3 4 5 6 7 8 9 10**

1. **تا چه اندازه میل به غذا خوردن دارید؟**

**خیلی کم خیلی زیاد**

|  |  |  |  |  |  |  |  |  |  |
| --- | --- | --- | --- | --- | --- | --- | --- | --- | --- |

**0 1 2 3 4 5 6 7 8 9 10**

1. **تمایل شما به خوردن مواد خوراکی شیرین چقدر است؟**

**مایل به خوردن نیستم تمایل زیادی به خوردن دارم**

|  |  |  |  |  |  |  |  |  |  |
| --- | --- | --- | --- | --- | --- | --- | --- | --- | --- |

**0 1 2 3 4 5 6 7 8 9 10**

1. **تمایل شما به خوردن موارد خوراکی شور چقدر است؟**

**مایل به خوردن نیستم تمایل زیادی به خوردن دارم**

|  |  |  |  |  |  |  |  |  |  |
| --- | --- | --- | --- | --- | --- | --- | --- | --- | --- |

**0 1 2 3 4 5 6 7 8 9 10**

1. **تمایل شما به خوردن موتد خوراکی پر چرب چقدر است؟**

**مایل به خوردن نیستم تمایل زیادی به خوردن دارم**

|  |  |  |  |  |  |  |  |  |  |
| --- | --- | --- | --- | --- | --- | --- | --- | --- | --- |

**0 1 2 3 4 5 6 7 8 9 10**
